# Benchmarking macaque brain gene expression for horizontal and vertical translation

**DOI:** 10.1126/sciadv.ads6967

**Published:** 2025-02-28

**Authors:** Andrea I. Luppi, Zhen-Qi Liu, Justine Y. Hansen, Rodrigo Cofre, Meiqi Niu, Elena Kuzmin, Seán Froudist-Walsh, Nicola Palomero-Gallagher, Bratislav Misic

**Affiliations:** ^1^ Montréal Neurological Institute, McGill University, Montréal, QC, Canada.; ^2^ Department of Psychiatry, University of Oxford, Oxford, UK.; ^3^ St John’s College, University of Cambridge, Cambridge, UK.; ^4^ Paris-Saclay University, CNRS, Paris-Saclay Institute for Neuroscience (NeuroPSI), Saclay, France.; ^5^ Institute of Neuroscience and Medicine (INM-1), Research Centre Jülich, Jülich, Germany.; ^6^ Department of Biology, Centre for Applied Synthetic Biology, Concordia University, Montréal, QC, Canada.; ^7^ Department of Human Genetics, Rosalind and Morris Goodman Cancer Institute, McGill University, Montréal, QC, Canada.; ^8^ Bristol Computational Neuroscience Unit, Bristol University, Bristol, UK.; ^9^ C. and O. Vogt Institute for Brain Research, Heinrich-Heine-University Düsseldorf, Düsseldorf, Germany.

## Abstract

The spatial patterning of gene expression shapes cortical organization and function. The macaque is a fundamental model organism in neuroscience, but the translational potential of macaque gene expression rests on the assumption that it is a good proxy for patterns of corresponding proteins (vertical translation) and for patterns of orthologous human genes (horizontal translation). Here, we systematically benchmark regional gene expression in macaque cortex against (i) macaque cortical receptor density and in vivo and ex vivo microstructure and (ii) human cortical gene expression. We find moderate cortex-wide correspondence between macaque gene expression and protein density, which improves by considering layer-specific gene expression. Half of the examined genes exhibit significant correlation between macaque and human across the cortex. Interspecies correspondence of gene expression is greater in unimodal than in transmodal cortex, recapitulating evolutionary cortical expansion and gene-protein correspondence in the macaque. These results showcase the potential and limitations of macaque cortical transcriptomics for translational discovery within and across species.

## Teaser

Macaque cortical gene expression recapitulates human gene expression and macaque receptor density and microarchitecture.

## INTRODUCTION

The macaque is a foundational and widely used model organism in neuroscience (*1*–*7*). Animal models allow invasive tracing, imaging, and recording, as well as numerous experimental manipulations that would not be possible in humans, allowing scientists to address a wider range of scientific questions (*8*). Compared to rodents and other nonhuman primates (NHPs) such as marmosets, the macaque has several desirable features for studying human brain structure and function. These include shared evolutionary history, a gyrified cortex, and a more human-like behavioral repertoire including greater ability to exert cognitive control against distractors during cognitive performance (*1*, *3*). These similarities are grounded in the genetic relatedness between the two species (92% genetic alignment with *Homo sapiens*) (*9*). As a result, the macaque has been used to study the evolutionary and developmental origins of brain anatomy, cognition, and behavior (*10*), as well as the consequences of targeted genetic mutations in transgenic animals (*11*–*13*).

Recent advances in high-throughput sequencing make it possible to map spatial patterns of gene expression across the cortex of humans and other species. This is valuable because gene expression is heterogeneous across the cortex: Spatial patterns of gene expression provide a blueprint for the brain’s structural and functional architecture (*14*–*17*). Multiple reports have inferred transcriptional signatures of cell types (*17*–*20*), neurotransmitter receptors (*21*–*24*), laminar differentiation (*25*), cortical thickness (*17*, *26*), structural and functional connectivity (*27*–*29*), brain dynamics (*30*, *31*), cognitive specialization (*32*), development (*26*), and disease (*18*, *33*, *34*), among others (*35*). Key to this endeavor has been the development of comprehensive spatial transcriptomics datasets. However, until recently, databases of comprehensive cortical gene expression have been restricted to human (*15*) and mouse (*14*, *36*). An exciting recent development is the availability of regionally resolved transcriptomics for the macaque brain (*16*, *17*, *37*), providing an unprecedented opportunity to combine this species’ experimental accessibility and genetic similarity to humans (*38*).

Effective translational discovery from macaque gene expression depends on two fundamental questions. The first question is whether orthologous genes display similar spatial patterning across human and macaque cortex. In other words, can regionally specific gene expression findings in the macaque be extrapolated to the human? The second question is whether spatial patterns of protein-coding genes in the macaque are a good proxy for spatial patterns of protein density in the same species, given the numerous intervening steps between mRNA transcription and expression of the corresponding protein at its final location. In other words, if we know the regional expression pattern of a gene from the macaque brain, can we infer the regional distribution of the protein that this gene codes for? Ultimately, as the field embarks toward comprehensive transcriptional mapping of the macaque brain, it is necessary to benchmark both the horizontal translational potential of these datasets (from macaque gene expression to human gene expression) (*38*) and their vertical translational potential (from macaque gene expression to other modalities within the macaque brain) (*38*).

**Fig. 1.**
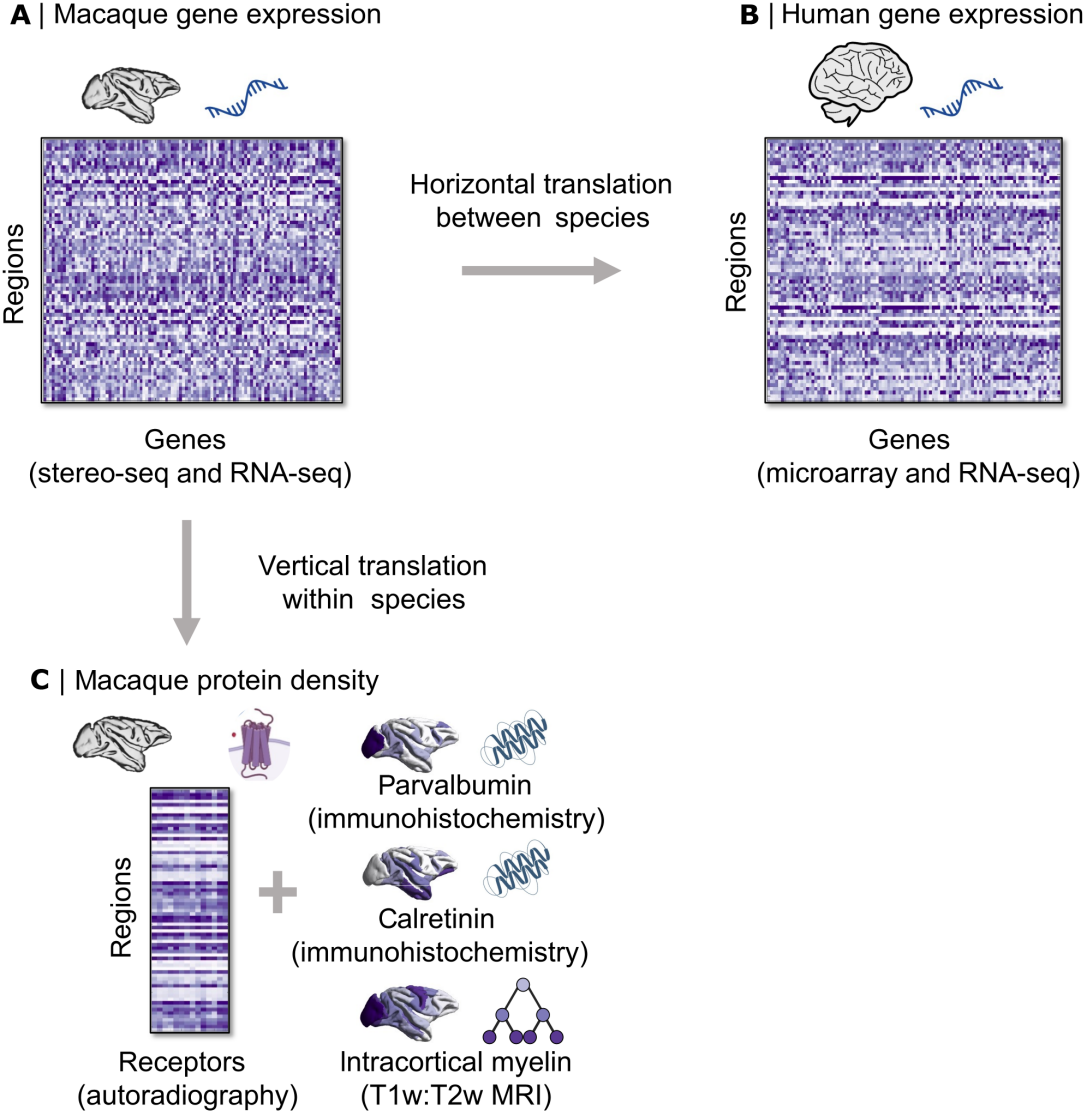
Benchmarking macaque cortical gene expression for horizontal and vertical translation. We compare macaque cortical gene expression from stereo-seq (*21*) (**A**) with (**B**) human gene expression from microarray in homologous regions (*20*) (horizontal translation between species). We also compare macaque cortical gene expression with (**C**) macaque protein density of receptors quantified from autoradiography (*50*), as well as in vivo intracortical myelination from T1w:T2w MRI ratio, and parvalbumin and calretinin density from immunohistochemistry (vertical translation within the macaque).

Here, we address these questions by analyzing a recently released database of *Macaca fascicularis* cortical gene expression (*16*) from high-resolution, large–field of view spatial transcriptomics [spatiotemporal enhanced resolution omics-sequencing (stereo-seq)] (*36*). To benchmark horizontal translation from macaque to human, we compare macaque stereo-seq transcriptomics with human cortical gene expression from postmortem microarray data of six adult donors’ brains, made available by the Allen Human Brain Atlas (AHBA) (*15*, *39*). To benchmark vertical translation between different data modalities within the macaque, we compare macaque cortical gene expression (*16*) against measurements of receptor density in the cortex of *M. fascicularis* obtained from postmortem autoradiography (*40*) (Fig. 1). Specifically, we focus on neurotransmitter receptors, a class of protein complexes that are essential for brain function, and therefore of particular interest in both basic research and clinical applications. Assessing the correspondence between gene expression and receptor density is of particular interest because gene expression is often used as a proxy for receptor density in the brain (*22*–*24*, *41*–*46*). However, measurements of gene expression and protein density do not always align (*47*–*54*). In the present report, we address these fundamental questions by assessing the extent to which gene expression in the macaque cortex reflects human gene expression, and the extent to which it reflects protein availability in macaque.

## RESULTS

We analyze three openly available datasets:

1. Macaque cortical stereo-seq gene expression (*16*).

2. Macaque cortical receptor density per neuron from in vitro autoradiography (*40*).

3. Human cortical microarray gene expression (*15*).

We further validate our results using measures of in vivo intracortical myelination and ex vivo density of the calcium-binding proteins parvalbumin and calretinin for the macaque cortex. Results are also replicated using macaque bulk RNA sequencing (RNA-seq) measures of gene expression. To obtain a common space for comparison, we apply the canonical Regional Mapping (RM) parcellation of the macaque cortex developed by Kötter and Wanke (*55*, *56*), alongside its recent translation to the human brain (*57*), allowing us to obtain a one-to-one mapping between regions in the two species (fig. S1; see Materials and Methods for details). We then ask two questions. First: what is the correspondence between macaque gene expression and neurotransmitter receptor density across cortical areas? Second: what is the correspondence between macaque cortical gene expression and human cortical gene expression?

### Gene-receptor correspondence in the macaque cortex

To determine whether gene expression is a suitable proxy for receptor density per neuron across macaque cortex, we compare stereo-seq macaque gene expression with quantitative measurements of corresponding receptor density from in vitro autoradiography, which uses radioactive ligands to quantify the endogenous receptors bound in the cell membrane (*16*, *40*). We focus on 13 receptors, covering both ionotropic and metabotropic receptors and spanning six neurotransmitter systems: noradrenergic (α_1_, α_2_), serotonergic (*5HT*_1A_, *5HT*_2A_), dopaminergic (*D*_1_), cholinergic (*M*_1_, *M*_2_), glutamatergic (*AMPA*, *kainate*, and *NMDA*), and GABAergic (*GABA*_A_, *GABA*_B_, and *GABA*_A/BZ_).

Neurotransmitter receptors can be classified as ionotropic (if signal transduction is mediated by ion channels) or metabotropic (G protein coupled). Ionotropic receptors are multimeric protein complexes, meaning that they consist of multiple subunits bound in the cell membrane, each encoded by a distinct gene. Metabotropic receptors are monomeric complexes: There is only a single protein embedded in the membrane, which is therefore encoded by a single gene. For monomeric receptors, we correlated regional protein density (from autoradiography) with regional expression of the corresponding gene (from stereo-seq). For multimeric receptors, in our main analysis, we show correlations between regional receptor density and regional expression of genes coding for the same receptor subunits as used in (*47*) (see Materials and Methods for details). The complete set of genes related to receptor subunits is shown in fig. S2.

**Fig. 2.**
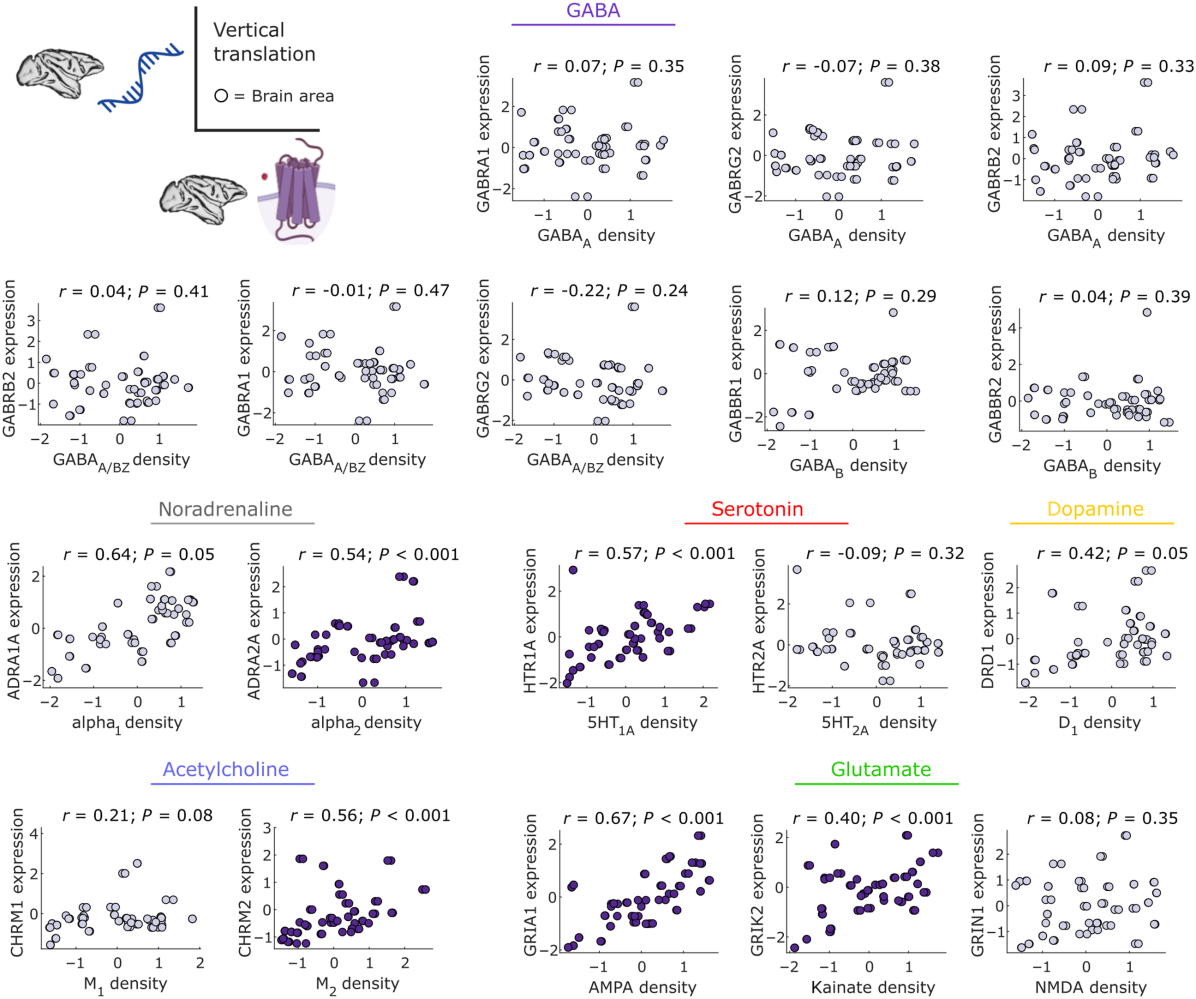
Vertical translation: Correlations between receptor density and corresponding gene expression in the macaque cortex. Only a few neurotransmitter receptors exhibit significant regional correlations with their corresponding gene across macaque cortical regions. Abscissa: regional receptor density from autoradiography (*50*). Ordinate: regional gene expression from stereo-seq (*21*). Each data point is a region of the Regional Mapping (RM) macaque cortical atlas. Indigo scatter plots indicate significant positive gene-receptor correlation (Spearman’s *r*, *P* < 0.05 against a null distribution of surrogate cortical maps with preserved spatial autocorrelation). Values are *z*-scored. See fig. S2 for results across the entire set of genes and receptors, and fig. S3 for results after adjustment for multiple comparisons.

Figure 2 shows the regional correlation between receptor density per neuron (*x* axis) and gene expression (*y* axis) for each receptor (i.e., each data point is one region of the RM atlas of macaque cortex). Because the brain exhibits nontrivial levels of spatial autocorrelation, traditional significance testing is not appropriate due to the inflated rate of false positives (*58*). Therefore, we assess the statistical significance of correlations between brain maps, against null distributions of surrogate brain maps that preserve the level of spatial autocorrelation in the data, generated using Moran Spectral Randomization (*59*) (see Materials and Methods for details). Throughout the article, significant associations (*P* < 0.05) are shown in indigo.

We find that a modest proportion of macaque neurotransmitter receptors correlate significantly with the expression of their main corresponding genes (5 out of 18 or 28%; Fig. 2), which is somewhat improved when considering the full set of receptor subunits for glutamate and GABA (24 out of 55, or 43.6%; fig. S2). However, the proportion of significant correlations remain around 30% after adjusting for multiple comparisons across all genes that are correlated with the same receptor (fig. S3). The observation of variable gene-receptor correspondence is consistent with findings in humans, using both in vivo positron emission tomography (PET) and postmortem autoradiography (*47*, *48*, *50*–*54*).

For receptors pertaining to neuromodulatory systems, we observe significant correlations between density per neuron of the adrenergic α_2_ receptor with *ADRA2A* gene expression; serotonergic *5HT*_1A_ receptor density and *HTR1A* gene expression; and muscarinic acetylcholine receptor *M*_2_ and *CHRM2* gene expression. Notably, *DRD1* gene expression barely fails to meet the threshold for a statistically significant correlation with dopamine *D*_1_ receptor density, after controlling for spatial autocorrelation (*P* = 0.05); similarly, α_1_ receptor density and *ADRA1A* gene expression exhibit the second-highest value of correlation (Spearman’s *r* = 0.64), albeit barely failing to reach significance after accounting for spatial autocorrelation (*P* = 0.05). For glutamate receptors, we observe significant correlations between *AMPA* receptor density and *GRIA1*, *GRIA3*, and *GRIA4* gene expression; kainate receptor density and *GRIK1*, *GRIK2*, and *GRIK3* gene expression; *NMDA* receptor density and *GRIN2B*, *GRIN2C*, and *GRIN3A* gene expression. For GABA receptors, we observed that *GABRA2*, *GABRA5*, *GABRB3*, and *GABRG1* exhibited significant correlations with both *GABA*_A_ receptor density and *GABA*_A/BZ_ binding site density, with additional significant correlations for *GABRA4*, *GABRA6*, and *GABRQ* (fig. S2).

Notably, the correspondence between *5HT*_1A_ receptor density and *HTR1A* gene expression had also been observed in humans with both in vivo PET and ex vivo autoradiography, by multiple studies (*47*, *48*, *52*, *54*), and even between human genes and macaque receptors (*40*). Altogether, the overall level of correspondence between gene expression and receptor density is consistent with recent findings in the human brain (*47*, *48*, *50*, *52*, *53*).

One potential reason why receptor density may not align perfectly with gene expression is that gene transcription occurs in the soma, whereas many receptors are expressed at the synapse, which may be far away, possibly even in a different brain area for neurons with long axons. To investigate this possibility, we correlate the receptor density of each region *A*, with the weighted average gene expression of the regions that *A* is directly structurally connected to, where the weights are the strengths of the connections from *A* to its neighbors (see Materials and Methods) (*34*). The rationale for this approach is that if receptors expressed in region *A* are the result of genes transcribed at the other end of long-range axons coming from other regions, then the receptor’s density in *A* should be better predicted by considering the average of gene expression in *A*’s neighbors. However, our results rule out this possibility (fig. S4). No additional significant gene-receptor correlations emerge, and the only significant correlations are between *GABRA6* gene expression and *GABA*_A_ receptor density, with *P* = 0.06 for the correlation between *HTR1A* gene expression and *5HT*_1A_ receptor density (fig. S4)—both of which were also significant in the original analysis.

Receptor expression can also vary depending on cell types. The relative prevalence of different cell types in each cortical region may therefore influence its profile of receptor expression. Chen *et al.* (*16*) provide a list of 23 transcriptomically derived cell types for the macaque cortex, based on data-driven clustering of their gene expression data. They comprise 10 subclasses of excitatory (glutamatergic) neurons, 7 subclasses of GABA-ergic neurons, and 6 subclasses of nonneuronal cells. We therefore use these data to ask whether cell subclasses have preferential correspondence with specific receptors, across the macaque cortex. Five out of six nonneuronal cell subclasses [all except vascular leptomeningeal cells (VLMCs)] exhibit preferential spatial colocalization with serotonin receptors across cortical regions. For oligodendrocytes, the greatest regional association is with the *5HT*_2A_ receptor. For oligodendrocyte precursor cells, endothelial cells, and especially microglia and astrocytes, the greatest correspondence is with the *5HT*_1A_ receptor (fig. S5). We find that L34 and L345 excitatory neurons and PV interneurons have the highest correspondence across regions with the *5HT*_2A_ receptor (fig. S5), which is known to be prominently expressed in layer 5 pyramidal neurons, but also found in excitatory neurons of layers 2 and 3 and parvalbumin-expressing interneurons (*60*, *61*). Likewise, astrocytes express *5HT*_1A_ (*62*) and α_1_ receptors (*63*), which we found to be the first and second most-associated receptors for this cell class (fig. S5). Altogether, these results at the level of macroscopic brain areas are consistent with microscale evidence about the preferential expression of different receptors across cell types.

### Laminar specificity of macaque gene-receptor correspondence

Different neuron types can be preferentially localized in specific cortical layers. Therefore, we next seek to determine whether the imperfect gene-receptor correlations could be attributable to layer-specificity. Because Chen *et al.* (*16*) provide gene expression data for each cortical layer, we can repeat our analysis of the regional correspondence between genes and receptors across the macaque cortex, but this time, using layer-specific gene expression data. Note that the macaque receptor density maps from the work of Froudist-Walsh *et al.* (*40*) do not provide layer-specific information. Rather, a single value of receptor density per region is available for each receptor. Therefore, for this analysis, we do not compare gene layer 1 with receptor layer 1, gene layer 2 with receptor layer 2, and so on. Rather, the same regional pattern of receptor density (without layer specificity) is compared against each layer-specific pattern of regional gene expression (figs. S6 to S11).

**Fig. 3.**
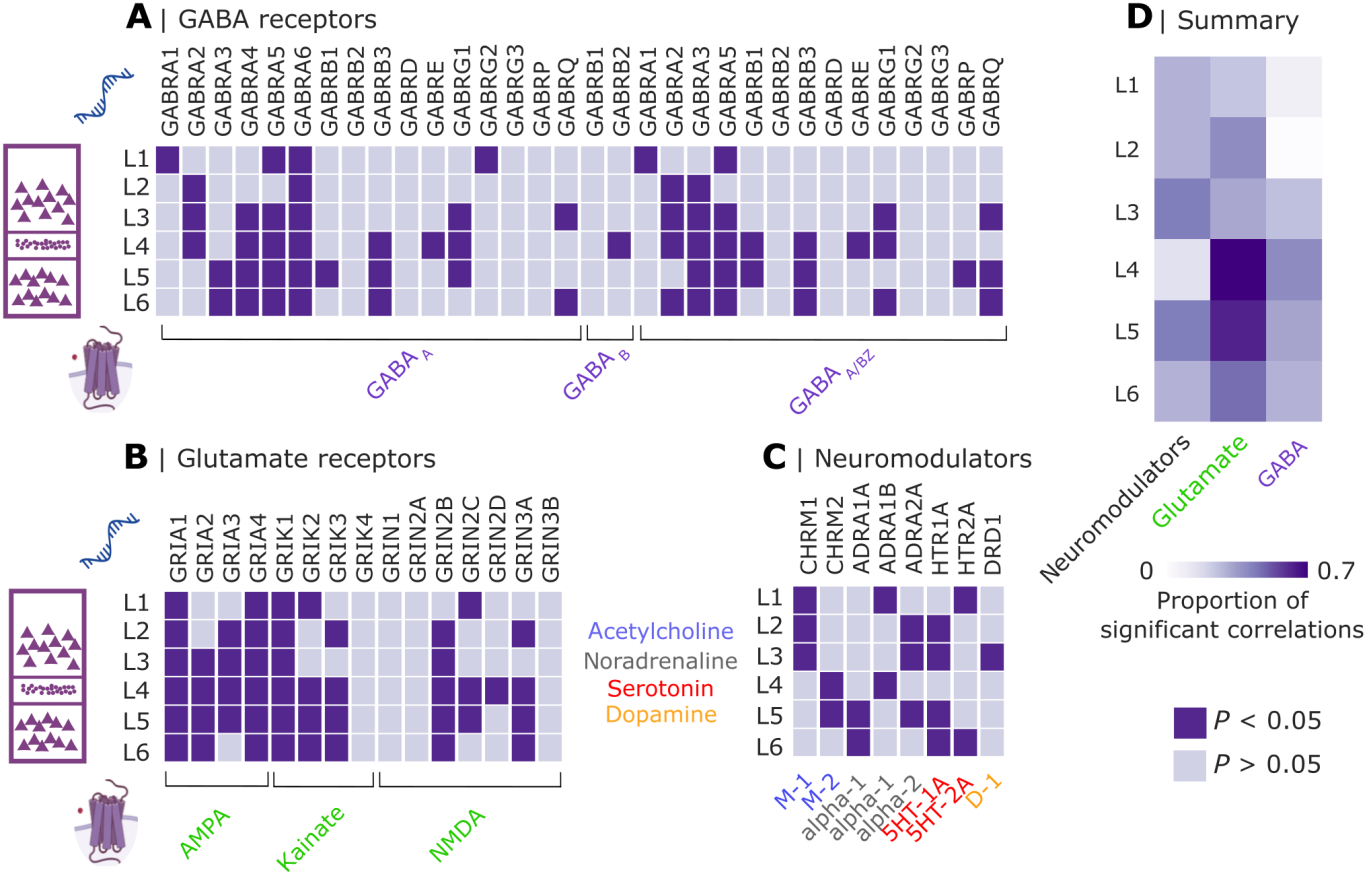
Correspondence between macaque gene expression and macaque receptor expression across cortical layers and receptor types. (**A**) Significance of gene-receptor correlations for GABA receptors. (**B**) Significance of gene-receptor correlations for glutamate receptors. (**C**) Significance of gene-receptor correlations for receptors pertaining to neuromodulatory systems (acetylcholine, noradrenaline, serotonin, and dopamine). For (A) to (C), indigo cells indicate significant positive gene-receptor correspondence (*P* < 0.05 against a null distribution of surrogate cortical maps with preserved spatial autocorrelation); gray cells indicate no significance. Rows indicate cortical layers, and columns indicate gene-receptor pairs. (**D**) Summary of the proportion of significant correlations from (A) to (C), for each layer and each broad receptor type. See fig. S12 for results after adjustment for multiple comparisons.

We find that the majority of gene-receptor pairs exhibit significant region-by-region correlation across at least one layer: 42 out of 55 (76%). Of these 42 significant gene-receptor pairs, 32 (58% of the total) have significant correlations for two or more layers. Crucially, the distribution is not uniform across layers, with deeper layers enjoying relatively higher proportion of significant correlations, especially for glutamate receptors (Fig. 3D). Notably, differences also emerge among receptor types, with the proportion of (uncorrected) significant correlations ranging from 31.2% of all layer-wise gene-receptor pairs for GABA to 41.6% for receptors pertaining to neuromodulatory systems and 53.3% for glutamatergic receptors (a significantly higher proportion than for GABA: χ^2^ = 12.65; *P* < 0.001). Upon adjusting for multiple comparisons across six layers and across all genes that are matched with the same receptor, we find that differences between receptor systems are amplified (fig. S12): 42.2% of glutamatergic gene-receptor pairs remain significant, which is a significantly higher proportion than for neuromodulatory receptors (25%; χ^2^ = 4.02; *P* < 0.045) and GABA-ergic receptors (6.77%; χ^2^ = 51.98; *P* < 0.001). The proportion of significant false discovery rate (FDR)–corrected gene-receptor correlations for GABA receptors is also lower than that for neuromodulatory receptors (χ^2^ = 13.67; *P* < 0.001). These results show that gene-protein correspondence is both layer–dependent and variable across neurotransmitter systems, being most robust for glutamate.

One limitation of the previous analysis is that layer-specific data were only available for gene expression, but not for receptor density, because Froudist-Walsh *et al.* (*40*) did not provide layer-specific receptor density for each macaque cortical region. However, we obtained layer-specific macaque receptor density for visual area V1 and for six subregions of the inferior parietal lobe (*64*), four of which (PF, PFG, PG, Opt) are also available in the database of layer-specific gene expression from (*16*). Note that no mapping to the RM atlas was required for this analysis, because both gene expression and receptor density data were available for each region in the original format. For each of these five regions (V1 and four IPL subregions), we can therefore expand on our previous layer-specific analysis in two complementary ways. First, we correlate the gene expression in each layer, against the corresponding receptor density in each layer, within each region (i.e., each layer is one data point). In line with our main analysis across cortical regions, we find that gene-receptor correlations across cortical layers exhibit greater magnitude for glutamate and neuromodulatory receptors than for GABA receptors, and they also appear greater for IPL subregions than V1 (fig. S13). Second, we compare the relative expression of all receptors in each layer against the relative expression of the corresponding genes in each layer (i.e., each data point is a gene-receptor pair). This analysis addresses the fact that neurons can be spatially extended across different layers of the same region. For example, deep-layer neurons may express genes whose corresponding receptor is found on distal tuft dendrites in layer 1. Notably, most significant FDR-corrected cross-layer correlations that we find are in V1, and they include both positive and negative correlations, such that greater gene expression in some layers (e.g., L2 and L5) corresponds to systematically lower receptor density in other layers (L4 and L1; fig. S14). Altogether, these layer-specific patterns of correspondences between genes and receptors showcase the intertwined nature of cortical chemoarchitecture and laminar differentiation.

### Macaque gene expression recapitulates microarchitectural features

We further assess whether our stereo-seq data allow us to recover gene-protein correlations that we should expect to observe, based on the literature. Notably, Fulcher *et al*. (*44*) reported high within-species correspondence in the mouse between *Pvalb* gene expression and the density of neurons expressing parvalbumin (the protein that *Pvalb* codes for), as well as high correspondence between mouse *Pvalb* and *Calb2*, and human *PVALB* and *CALB2* gene expression. In addition, Burt *et al.* (*42*) observed that macaque protein density of the calcium-binding proteins parvalbumin and calretinin from immunohistochemistry exhibits similar spatial patterns to the expression of human *PVALB* and *CALB2* genes, which code for these proteins in humans. Together, these previous results suggest that we should expect to observe similar regional patterns for macaque *PVALB* gene expression and parvalbumin protein density, and for macaque *CALB2* expression and calretinin protein density. Observing such a correspondence would corroborate the quality of the stereo-seq gene expression data.

**Fig. 4.**
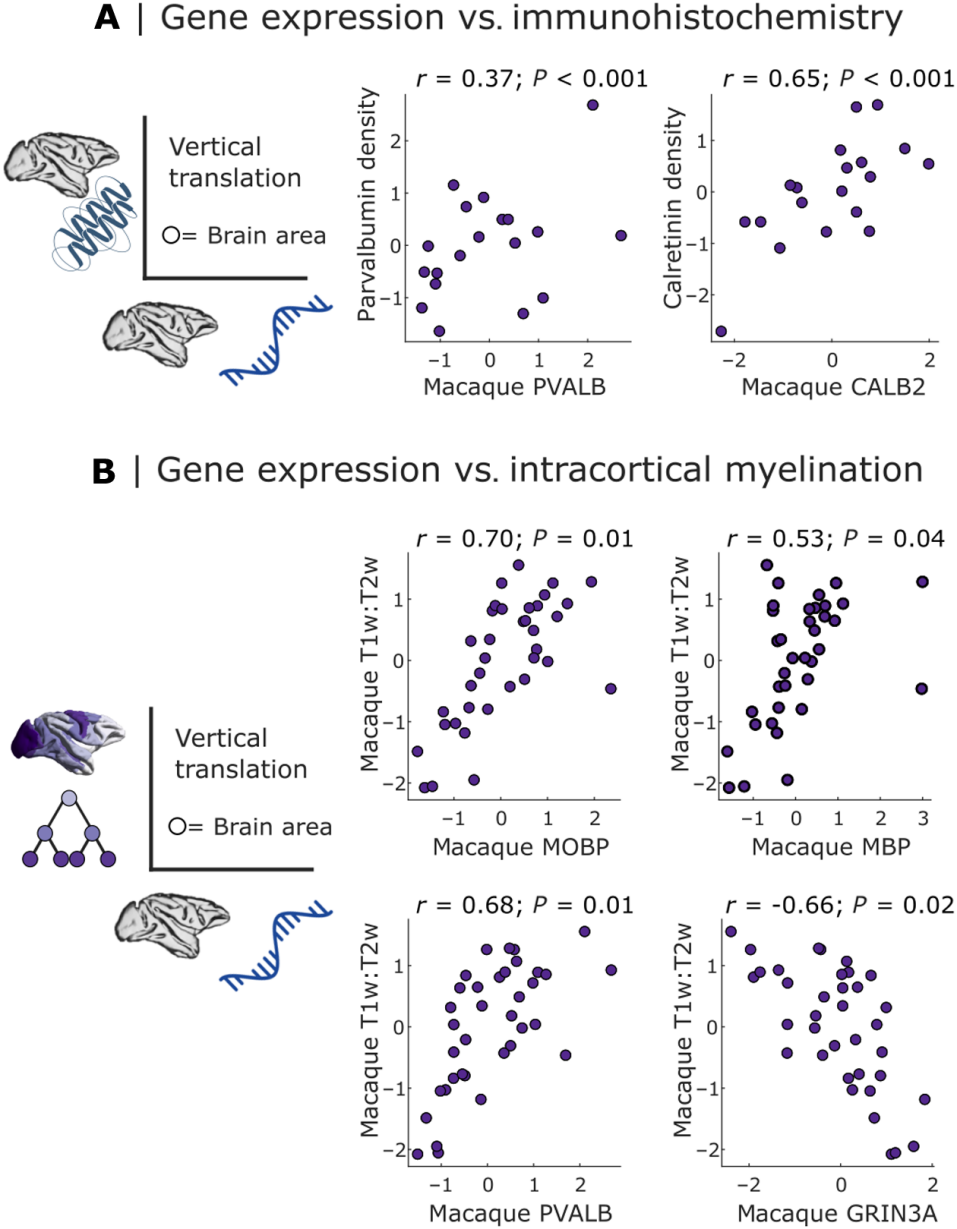
Macaque gene expression recapitulates microarchitectural features. (**A**) Region-by-region correspondence of macaque PVALB and CALB2 gene expression, with the immunohistochemically derived regional density of the proteins that these genes code for: parvalbumin and calretinin, respectively. (**B**) Region-by-region correspondence of macaque gene expression with intracortical myelination from in vivo T1w:T2w MRI ratio. Indigo scatter plots indicate significant region-by-region correspondence (Spearman’s *r*, *P* < 0.05 against a null distribution of surrogate cortical maps with preserved spatial autocorrelation). Each data point is a region of the RM macaque cortical atlas. Values are *z*-scored.

We show that macaque *PVALB* gene expression from stereo-seq is significantly correlated (*r* = 0.37, *P* < 0.001) with an independent measure of density of parvalbumin-immunoreactive interneurons from immunohistochemistry (*65*–*68*) assembled and made available by Burt *et al.* (*42*) (Fig. 4A). Likewise, macaque *CALB2* gene expression from Stereo-seq is significantly spatially correlated with density of calretinin-immunoreactive interneurons in the macaque cortex (*42*) (*r* = 0.65, *P* < 0.001; Fig. 4A). We replicate these results using the relative prevalence of calretinin-immunoreactive and parvalbumin-immunoreactive neurons across a subset of visual, auditory, and somatosensory regions of the macaque from (*68*), one of the original studies aggregated by Burt and colleagues (fig. S15).

In addition, Fulcher *et al*. (*44*) reported that both humans and mice exhibit significant spatial correspondence between in vivo intracortical myelination [estimated from the ratio of T1-weighted to T2-weighted magnetic resonance imaging (MRI) signal] and expression of the key myelin-related genes *MBP/Mbp* and *MOBP/Mobp*, as well as *PVALB/Pvalb* and *GRIN3A/Grin3a*. Here, we demonstrate that each of these relationships is also observed in the macaque: We find significant positive region-by-region correlations between macaque intracortical myelination (T1w:T2w ratio) and macaque cortical expression of *MOBP* (*r* = 0.70, *P* = 0.01), *MBP* (*r* = 0.53, *P* = 0.043), and *PVALB* (*r* = 0.68, *P* = 0.01), as well as a significant negative association between T1w:T2w ratio and *GRIN3A* gene expression (*r* = −0.66, *P* = 0.025) (Fig. 4B)—precisely as reported in (*69*). In addition to demonstrating a close correspondence across cortical regions between ex vivo expression of myelin-related genes and an in vivo marker of cortical myelination in the macaque brain (*70*–*72*), these results also demonstrate close alignment of our results with two different mammalian species: human and mouse. Collectively, despite moderate alignment across cortical regions in the specific case of gene-receptor correspondence, these results indicate that macaque stereo-seq gene expression can recapitulate many other in vivo and ex vivo features of macaque cortical microarchitecture.

### Cross-species correspondence of gene expression

Having assessed the potential of macaque gene expression for vertical translation (within-species), we next proceed to assess the translational potential of macaque gene expression to human (horizontal translation). We compare macaque regional patterns of gene expression from stereo-seq, against regional patterns of human gene expression obtained from the AHBA microarray data (*15*). In addition to the list of genes coding for receptor subunits already included in the previous analyses (*47*, *48*), we also include the list of brain-related genes examined by Fulcher *et al.*’s (*44*) investigation of interspecies correspondence of gene expression between mouse and human. This list includes not only genes coding for neurotransmitter receptors but also neuropeptide receptors, interneuron cell-type markers (parvalbumin, somatostatin, calbindin, and vasoactive intestinal polypeptide), and the four most abundant mRNAs in myelin (*MBP*, *FTH1*, *PLEKHB1*, and *MOBP*) (*44*). For all analyses, we only consider genes that (i) have a macaque ortholog and (ii) are available in both the human and macaque datasets after accounting for all preprocessing criteria, such as intensity filtering, yielding a total of 99 genes.

**Fig. 5.**
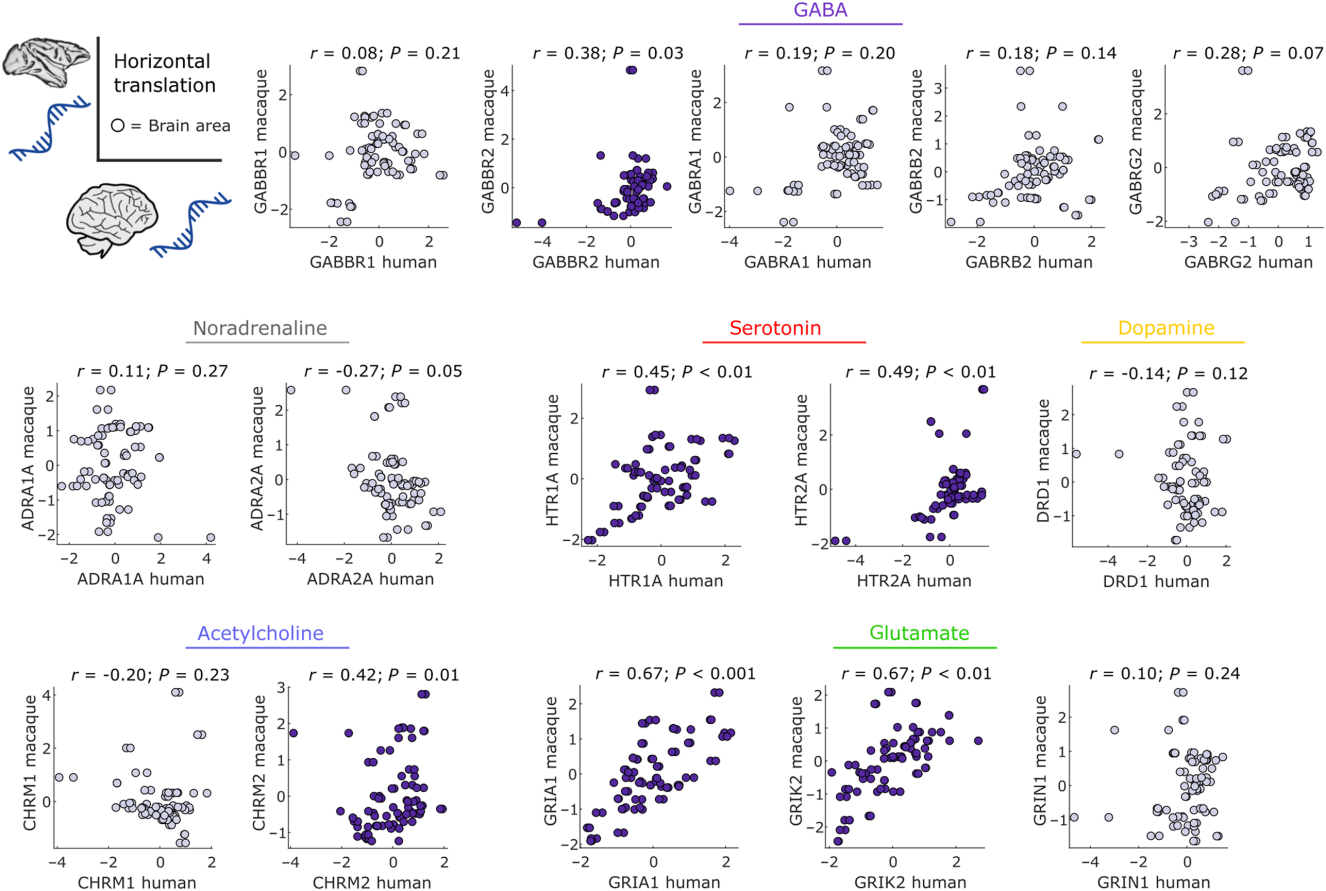
Horizontal translation: Regional correspondence between the expression of the main available brain-relevant genes in humans (from microarray) and macaques (from stereo-seq). The majority of macaque genes exhibit significant regional correlation with the corresponding human ortholog genes. Indigo scatter plots indicate significant human-macaque correspondence (Spearman’s *r*, *P* < 0.05 against a null distribution of surrogate cortical maps with preserved spatial autocorrelation). Each data point is a region of the Regional Mapping (RM) macaque cortical atlas. Values are *z*-scored.

We find that 53 of 99 (53.5%) genes considered exhibit significant region-to-region correlation between humans and macaques, after accounting for spatial autocorrelation. A representative sample of cross-species gene correlations pertaining to receptors is shown in Fig. 5; the full set is shown in figs. S16 and S17. In other words, we find that interspecies correspondence of gene expression for genes pertaining to cell-type markers and receptor subunits is greater than the correspondence between macaque gene expression and density of the corresponding receptors, as shown in the previous section. However, the proportion of significant interspecies correlations of gene expression (53%) is comparable to the proportion of macaque gene-receptor pairs having more than one significant correlation across cortical layers (58%).

**Fig. 6.**
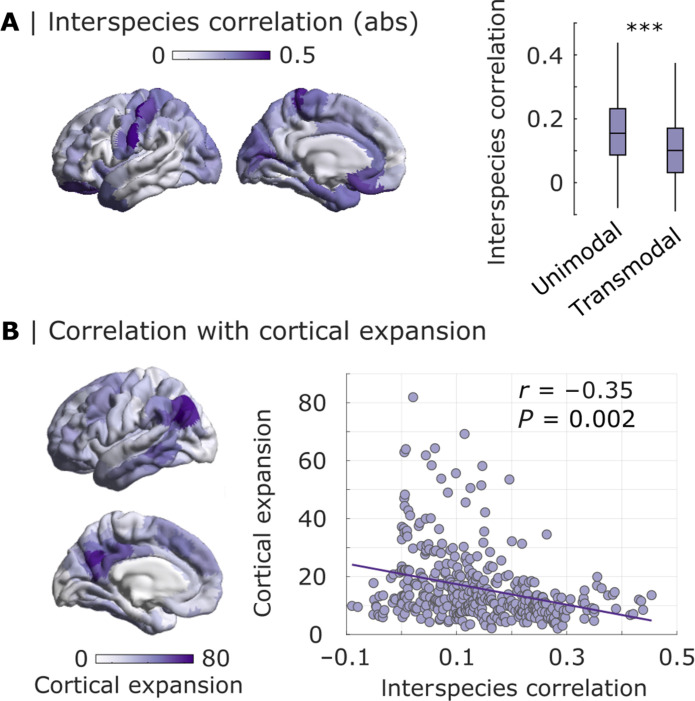
Regional correlation between human and macaque patterns of cortical gene expression recapitulates cortical expansion. (**A**) Intensity of color of each region indicates the magnitude of Spearman’s correlation between human microarray and macaque stereo-seq gene expression for that region, across genes. Data are re-parcellated from the human version of the Regional Mapping (RM) atlas to the Schaefer-400 parcellation. The interspecies correlation of regional gene expression is significantly higher in unimodal than transmodal regions; unimodal mean = 0.16; transmodal mean = 0.11; *t*_398_ = 5.20, Cohen’s *d* = 0.52, *P* < 0.001 from independent-samples *t* test. (**B**) The cortical pattern of regional interspecies correlations of gene expression is significantly negatively correlated with the spatial pattern of cortical expansion from macaque to human from (*90*) (each data point is a region of the Schaefer cortical atlas); Spearman’s *r* = −0.35, *P* = 0.002 after accounting for spatial autocorrelation.

Is interspecies gene expression correspondence uniform across the brain, or is it regionally heterogeneous? Complementary to correlating expression of each gene across regions (i.e., each data point being a cortical region), we can also consider the correlation of expression of different genes at each cortical region (i.e., with data points being genes) (Fig. 6A). We find that the regional interspecies correlation of genes is significantly greater in the unimodal than in the transmodal regions of the cortex (Fig. 6A). These systematic differences between gene expression in the two species are reflected in regionally heterogeneous phylogenetic divergence between humans and NHPs, including macaques (*73*–*78*). Cortical regions that exhibit the lowest correspondence of gene expression between macaque and human are also the regions that have undergone the greatest cortical expansion between the two species, as quantified in a recent report of human-macaque cortical expansion by Xu *et al.* (*77*). Specifically, there is a significant negative correlation between the two regional patterns: Spearman’s *r* = −0.35, *P* = 0.002 (Fig. 6B).

We can also adopt the same approach to estimate region-by-region correspondence between gene expression and protein density in macaque cortex. In addition to receptors, we also consider intracortical myelin from T1w:T2w ratio [matched with *MBP*, *MOBP*, *PLEKHB1*, and *FTH1* genes as per Fulcher *et al*. (*44*); calretinin (matched with *CALB2*) and parvalbumin (matched with *PVALB*)]. This cortical map shows the greatest gene-protein correspondence in unimodal (visual and somatomotor) regions (fig. S18), and it converges with the map of regional interspecies correspondence of gene expression (Spearman’s *r* = 0.43, *P* < 0.001; fig. S19). Thus, both horizontal and vertical translation of macaque gene expression exhibit systematic regional heterogeneity, being most pronounced in unimodal cortices.

### Correspondence between macroscopic cortical gradients

So far, we considered all correspondences on an individual gene and protein basis. However, a rich literature posits that much of the region-by-region variation in gene expression follows a small set of dominant macroscale patterns or “gradients,” often identified by applying dimensionality reduction techniques such as principal components analysis (PCA) (*15*, *42*, *44*, *79*). We therefore ask whether the results that we observed so far (moderate correspondence between gene expression and protein density in the macaque; greater correspondence between gene expression in the macaque and gene expression in human) can also be observed at the level of multivariate gradients.

**Fig. 7.**
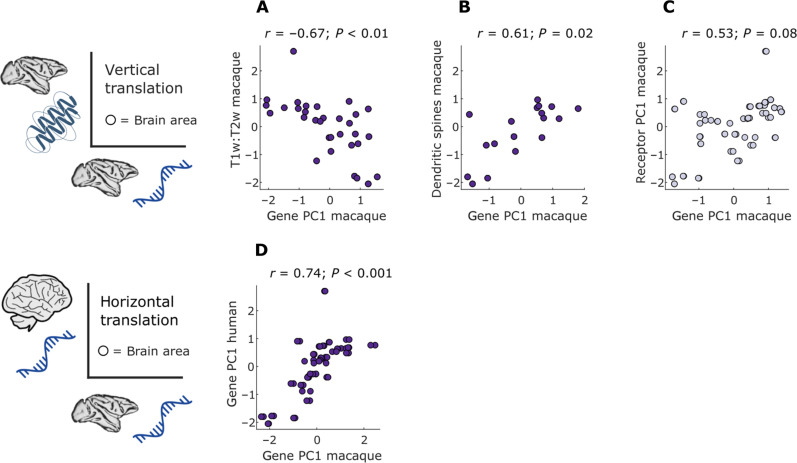
Horizontal and vertical translation of macaque cortical gradients. (**A**) Macaque gene PC1 is significantly correlated with macaque intracortical myelination (quantified from T1w:T2w ratio). (**B**) Macaque gene PC1 is significantly correlated with the number of dendritic spines of layer 3 pyramidal neurons. (**C**) Macaque gene PC1 does not correlate with macaque receptor PC1 after accounting for the role of spatial autocorrelation. (**D**) Macaque gene PC1 is significantly spatially correlated with human gene PC1. Each data point is a region of the RM macaque cortical atlas. Indigo scatter plots indicate significant regional correlation (Spearman’s *r*, *P* < 0.05 against a null distribution of surrogate cortical maps with preserved spatial autocorrelation). Values are *z*-scored.

Starting from macaque gene PC1 (the regional pattern that captures the most variance in the region-by-gene matrix, as obtained from PCA), we show that it exhibits a significant region-by-region correlation with macaque in vivo intracortical myelination quantified from T1w:T2w MRI ratio (*r* = −0.67, *P* < 0.01; Fig. 7A). This is what we should expect, based on previous results of correlations between gene PC1 and T1w:T2w ratio in both humans and mice (*42*, *44*). We replicate this result using a different dataset of macaque intracortical myelination for a subset of regions (fig. S20). These results provide further biological validation for our gene expression data.

Burt *et al.* (*42*) had also shown that in the macaque, T1w:T2w ratio is negatively correlated with the number of dendritic tree spines of layer 3 pyramidal neurons. Taking together this finding and the finding that T1w:T2w ratio is negatively correlated with gene PC1 across species, we should expect a positive correlation between macaque gene PC1 and macaque regional L3 dendritic spine count. However, this relationship has not been demonstrated before. This is indeed what we find: Macaque gene PC1 is positively correlated across regions with the regional dendritic spine count, *r* = 0.61, *P* = 0.02 (Fig. 7B). Together, these correspondences of macaque gene PC1 with independent in vivo and ex vivo measures in the macaque brain provide convergent corroboration for the validity of the gene expression data used here.

However, in line with the moderate pairwise correspondence between macaque genes and receptors that we observed, the macaque gene PC1 narrowly fails to exhibit significant region-by-region correlation with the principal component of macaque receptor density from autoradiography (receptor PC1), once spatial autocorrelation is taken into account (Spearman’s *r* = 0.53, *P* = 0.08; Fig. 7C). In contrast, we find a strong significant region-by-region correlation between macaque gene PC1 and human gene PC1—despite being obtained from different techniques, namely, stereo-seq for the macaque and microarray for the human data (*r* = 0.74, *P* < 0.001; Fig. 7D). This result corroborates the significance of correlations between numerous individual genes across the two species and provides further evidence for interspecies correspondence of gene expression between macaque and human.

### Replication with macaque RNA-seq gene expression

Although the stereo-seq database of macaque cortical gene expression is a valuable resource, one potential limitation is that stereo-seq has relatively low gene coverage from single cells (*16*). Therefore, to ensure the robustness of our results, we replicate them using an independent dataset of macaque region-specific gene expression from bulk RNA-seq (*17*). These data comprise a smaller set of 18 genes coding for neurotransmitter receptors, transporters, and synthesis. Among these, 17 are also present in the stereo-seq dataset from (*16*) (see Materials and Methods). We find that 11 of 17 genes (64%) exhibit significant region-by-region correlations across macaque cortex between stereo-seq and bulk RNA-seq, including cholinergic *CHRNA1*, dopaminergic *DRD2*, glutamatergic *GRIA4*, GABA-ergic *GABRQ*, and serotonergic *HTR2C* (fig. S21).

Of the 18 macaque genes shared by Bo *et al.* (*17*), 11 are also available in our AHBA microarray dataset. Replicating our stereo-seq results for macaque-human correspondence, we find several significant region-by-region correlations between macaque bulk RNA-seq gene expression and human microarray gene expression (fig. S22). Notably, a number of macaque bulk-RNA genes exhibited significant correlations both with macaque stereo-seq and with human microarray gene expression: *DDC*, *GLRA2*, *GRIA4*, *HTR2C*, and *PNMT*.

We also compare macaque RNA-seq gene expression against macaque receptor density. Because only a small number of macaque genes are available from Bo *et al.* (*17*), we compare these genes against all available macaque receptors pertaining to the same neurotransmitter systems (see Materials and Methods). Altogether, we find that 12 of 30 genes exhibit statistically significant cortical correlations with receptors pertaining to the same neurotransmitter system, including correlations between *AMPA* receptor and *GRIA4* gene expression, between *GABRQ* gene expression and *GABA*_A/BZ_ receptor density, and between α_2_ receptor and *ADRA2C* gene expression, providing validation for our main results from stereo-seq (fig. S23; see fig. S24 for FDR-corrected results). At the multivariate level, we find that macaque gene PC1 from bulk RNA-seq is significantly correlated with macaque gene PC1 from stereo-seq, and also with macaque receptor PC1 (fig. S25). Overall, the smaller list of macaque cortical gene expression maps provided by Bo *et al.* (*17*) produces similar patterns of spatial correspondence to those observed from macaque stereo-seq (*16*).

### Replication with human RNA-seq gene expression

We also replicate results using an alternative measurement of human gene expression, RNA-seq, available in two of six AHBA donors (*15*). Patterns of region-by-region correspondence between human RNA-seq gene expression and macaque stereo-seq gene expression are consistent with our previous results (where human gene expression was obtained from microarray), including significant correlations between human and macaque *PVALB*, *SST*, *CALB2*, *GRIA1*, *GRIK2*, and *HTR1A* (figs. S26 and S27). Univariate correspondences are also recapitulated at the multivariate level, where we replicate the significant correlation between macaque stereo-seq gene PC1 and human gene PC1 obtained from RNA-seq: Human gene PC1 from RNA-seq is significantly correlated both with macaque gene PC1 from stereo-seq and with macaque gene PC1 from RNA-seq (fig. S28). In other words, not only is macaque gene PC1 consistent across stereo-seq and bulk RNA-seq, but we also find that macaque gene PC1 and human gene PC1 are significantly correlated, regardless of which modality is used to measure macaque gene expression (stereo-seq or bulk RNA-seq) and regardless of which modality is used for human gene expression (microarray or RNA-seq).

Last, the pattern of interspecies correlation of each region’s gene expression, which we obtained by comparing macaque stereo-seq against human microarray data, is also recapitulated when human RNA-seq data are used instead (fig. S29). Collectively, these checks demonstrate that our results are robust across different datasets and measurements (human microarray and RNA-seq; macaque stereo-seq and RNA-seq).

## DISCUSSION

Comprehensive gene expression in the macaque holds promise as a means of learning about correspondence with the human brain and to expand the value of the macaque as a model organism, including for gene therapy (*12*, *13*).

Within-species correspondence between macaque gene expression and receptor density is generally moderate, and variable across receptor-gene combinations. This result is in line with the modest correspondence between mRNA and protein abundance previously reported in the literature (*80*–*83*), including in human brain tissue (*84*). Our results validate recent results pertaining to gene-receptor correspondence in the human brain. These studies showed that few receptors (whether measured from in vivo PET or postmortem autoradiography) exhibit significant regional covariation with expression of the corresponding genes from human microarray data (*47*, *48*, *50*, *52*, *53*). Nonetheless, we do find that some specific gene-protein pairs exhibit robust correlations across regions in the macaque cortex. These include parvalbumin-*PVALB* and calretinin-*CALB2* correspondence, consistent with the literature in both human and mouse (*42*, *44*). Likewise, we found significant region-by-region correlation between *HTR1A* gene expression and *5HT*_1A_ receptor density in the macaque. In the human, Hansen *et al.* (*47*) showed that the *HTR1A* gene was the only one to show significant correlation with expression of the corresponding receptor (*5HT*_1A_) across both PET and autoradiography. Correspondence between *HTR1A* mRNA and *5HT*_1A_ receptor density has been reported in humans by multiple studies (*48*, *52*, *54*, *85*, *86*). The present results corroborate the results obtained in (*47*, *48*, *50*) in a different species and with different modalities. They also validate the recent result of Froudist-Walsh *et al.* (*40*), who reported a significant correlation between human *HTR1A* gene expression and macaque *5HT*_1A_ receptor density. Likewise, Hansen *et al.* (*47*) and Murgaš *et al.* (*48*) reported significant correlation between *ADRA2A* and α_2_ in humans, and Froudist-Walsh *et al.* (*40*) reported significant correlation between macaque α_2_ density and human *ADRA2A* gene expression. Together, these previous results suggest that *ADRA2A* expression and α_2_ density might also be correlated in the macaque, which is precisely what we found. Although we observed limited gene-receptor correspondence in the macaque when considering gene expression aggregated across cortical layers, 76% of gene-receptor pairs exhibit a significant correlation for at least one cortical layer. This finding suggests that the limited gene-receptor correspondence reported in the human literature (*47*, *48*) may partly be due to the aggregation of gene expression across cortical layers. Thanks to a number of regions for which both gene expression and receptor density are available in each cortical layer, we uncovered complex gene-receptor relationships both across layers of the same region, and between different cortical layers. These laminar relationships further vary by regional identity and neurotransmitter system, highlighting the need for more comprehensive datasets of layer-specific receptor density.

More broadly, there are numerous biological reasons why expression of a gene may diverge from density of the corresponding protein (*87*). First, total gene expression levels ignore mRNA stability from posttranscriptional regulation, which affects protein abundance, as RNA levels depend on both transcription and decay rates (*88*). Second, protein abundance is influenced by variations in protein stability, half-life, and the activity of transport machinery that determines protein localization within the cell (*82*, *89*, *90*). Rates of degradation in postmortem tissue can vary widely for mRNA and proteins, spanning several orders of magnitude: minutes for mRNA and hours to years for proteins (*50*, *80*, *83*, *87*). Third, studies in yeast have demonstrated that translation rate also alters protein levels, in turn affecting the correlation between mRNA expression and protein abundance (*91*). Fourth, the protein itself may be transported far from the coding mRNA, resulting in a spatial discrepancy between protein levels, and gene expression quantified from mRNA abundance. For example, this could be the case for presynaptic receptors located at the end of long axonal projections. However, we largely ruled out this possibility by showing that receptor density of a region does not correlate with the weighted average gene expression of that region’s structurally connected neighbors.

Altogether, any of these biological processes may contribute to the observed difference in levels of mRNA expression and protein density in the macaque cortex, including both calcium-binding proteins and receptors. However, neurotransmitter receptors are protein complexes bound in the cell membrane. Therefore, there are additional receptor-specific biological processes that may explain why gene-receptor correspondence was generally not as good as the correspondence between gene expression and parvalbumin and calretinin. This is because there are multiple steps between synthesis of a single protein and that protein forming part of a multimeric structure embedded in the cellular membrane. For example, any formed receptors that do not bind to the membrane (such as receptors localized to intracellular compartments) will fail to be detected from autoradiography. In addition, posttranscriptional and posttranslational modifications (e.g., receptor assembly and trafficking) can further contribute to decoupling mRNA expression from the final receptor prevalence. Last, gene-receptor correspondence will also be influenced by variations in subunit composition of a receptor. Changes in subunit composition are to be expected, because they play an integral role in synaptic function and plasticity (*92*, *93*).

The correspondence between gene expression and protein density may also vary depending on the prevalence of different cell types (*94*). We find that cell types exhibit variable spatial associations with different receptors across regions of macaque cortex. In particular, all but one subtype of nonneuronal cells are preferentially spatially associated with serotonin receptors. Notably, there is mounting evidence that nonneuronal cells such as astrocytes, oligodendrocytes, and microglia are implicated in depression and other psychiatric disorders through their roles in monoamine reuptake and synaptic function (*95*, *96*), demyelination (*97*, *98*), and inflammation (*99*, *100*), respectively. The *5HT*_1A_ and *5HT*_2A_ receptors are both prominent targets for pharmacological interventions for mood disorders (*101*–*103*). The present results of an association between serotonin 1A and 2A receptors and support cells in macaque corroborate their respective involvement in mood disorders in the literature, which may merit further investigation in humans. In this context, it is promising that we found both robust within-species correspondence between *5HT*_1A_ receptor density and *HTR1A* gene expression, and robust correspondence of cortical *HTR1A* expression across macaque and human.

How the spatial transcriptomes of the macaque and human align across the cortical sheet is an important benchmark for the translational potential of this model organism. We find that more than half (53%) of genes included in the present study exhibit significant region-by-region correlation between human and macaque. Our finding of conserved gene expression across homologous macroscale regions of primate cortex complements the findings of Krienen *et al.* (*104*), who reported that cross-species correlations of RNA expression across homologous interneuron classes were more consistent among primates than in the primate–mouse or primate–ferret comparisons. We find that both horizontal and vertical translation of macaque gene expression are regionally heterogeneous but highly systematic. Namely, interspecies correspondence of gene expression is greatest in the unimodal cortex and lowest in the transmodal cortex. This result is consistent with Beauchamp *et al.* (*105*), who reported that the similarity of gene expression patterns between human and mouse is higher in sensorimotor than in association cortices. This pattern of genetic divergence may then drive divergence in cell types, circuits, and macroscopic features of cortical organization. For example, we find that regions in which human gene expression diverges most from macaque also show the greatest expansion in gray matter, as reported by Xu *et al.* (*77*). In addition, regions where there is better correspondence between human and macaque gene expression (greater horizontal translation) also show better gene-protein correspondence within the macaque (greater vertical translation). Thus, unimodal regions exhibit tighter tethering of protein density to gene expression within the same species, and more conserved gene expression across species, converging with evidence for more limited evolutionary divergence in unimodal cortices (*73*–*78*, *106*). This regional heterogeneity bears an important implication for future spatial transcriptomics studies; namely, confidence in translational inferences depends on where in the brain the transcriptome is sampled. Therefore, for horizontal translation from macaque to human, it is desirable that transcriptomic sampling should span a variety of brain regions (ideally, whole brain) and be accompanied by precise three-dimensional spatial locations, including laminar information.

Correspondence between human and macaque gene expression is also heterogeneous in terms of specific genes. Once again, however, this heterogeneity is not random. In contrast, several genes displaying high interspecies correspondence between human and macaque are also known to be conserved between humans and mice. Previous work identified a number of brain-related genes that exhibit strong interspecies consistency between human and mouse, using each species’ T1w:T2w ratio map to define a common spatial reference for interspecies comparison. These genes include the interneuron markers *PVALB/Pvalb* and *CALB2/Calb2*; the oxytocin receptor gene *OXTR/Oxtr*; the glutamate receptor genes *GRIN3A/Grin3a*, *GRIK1/Grik1*, *GRIK2/Grik2*, and *GRIK4/Grik4*; the myelin marker genes *MOBP/Mobp* and *MBP/Mbp*; and the serotonin *HTR1A/Htr1a* gene (*44*). Here, we find that except for *GRIK4* and *MBP*, all these genes exhibit significant correlations between human microarray and macaque stereo-seq data, with most (including *GRIK4*) also correlating with human RNA-seq data. Note that here we used direct region-to-region correlation between human and macaque cortical patterns, rather than the indirect approach of Fulcher *et al*. (*44*), thanks to the availability of the same parcellation in both species. Together with the results of Fulcher *et al*. (*44*), our results further establish that many genes pertaining to microstructure and receptors exhibit robust regional similarities across mammalian species, even when measured through diverse techniques (in situ hybridization in the mouse, microarray and RNA-seq in humans, and stereo-seq and RNA-seq in macaques).

This comparative work integrates multiple unique datasets, entailing inherent methodological limitations for each dataset and for comparisons between datasets. Specifically, limitations include the following: (i) macaque gene and receptor expression come from different animals; (ii) data are postmortem rather than in vivo, and the consequent small number of animals and humans that provided data; (iii) gene expression was estimated from different transcriptomics techniques in different species (microarray and RNA-seq for humans; stereo-seq and RNA-seq for macaques); (iv) limited cortical coverage for the macaque gene expression data (left hemisphere only); (v) limited cortical coverage for the autoradiography data (no temporal lobe); and (vi) restriction to cortical sampling.

In light of these limitations, we instituted several checks. As expected based on analogous correspondences in both human and mouse (*42*, *44*), we showed that the principal component of macaque gene expression correlates with macaque intracortical myelination from T1w:T2w MRI ratio (Fig. 7A), as well as dendritic spine density (Fig. 7B). Macaque gene PC1 also correlated with human gene PC1, regardless of how gene expression was quantified in either species, whereas the match with macaque receptor PC1 is imperfect—recapitulating in a multivariate fashion the results observed for individual genes and receptors. We also showed that macaque *PVALB* gene expression correlates with human *PVALB* expression. This interspecies correspondence corroborates the strong interspecies correspondence between human *PVALB* and mouse *Pvalb* (*44*)). Using an independent dataset, we further showed that macaque *PVALB* also correlates with parvalbumin protein density in the macaque. Together with the correspondence between *HTR1A* and *5HT*_1A_, this evidence indicates that when there is reason from the literature to expect a correspondence between protein density and gene expression, it is also observed in our data. In particular, the correspondence between *HTR1A* and *5HT*_1A_ was observed in both supragranular (L2 and L3) and infragranular (L5 and L6) layers of macaque cortex, consistent with human findings (*47*). This is noteworthy because our own layer-wise data were for the gene expression, not for the receptor density, whereas Hansen *et al.* (*47*) used layer-wise information about receptor density, but not about gene expression. Notably, *5HT*_1A_ receptors are expressed more prominently near the soma (including the axon initial segment), than in the apical dendrite (*107*), which may provide a neuroanatomical explanation for their higher correlation in layers 2, 3, and 5. In contrast, the high glutamatergic gene-receptor correlations for layer 4 may occur because L4 neurons do not have big dendritic trees (in particular, they do not have apical dendrites). L2/3 and L5 pyramidal cells have broad dendritic trees with apical dendrites reaching up to L1 and, therefore, can be expected to have many receptors far from the soma. Last, to ensure robustness and mitigate the limitations of stereo-seq such as low gene coverage from single cells, we replicated our results using a bulk RNA-seq macaque gene expression dataset, different protein measurements, and different human gene expression data (bulk RNA-seq instead of microarray).

As a final limitation, the datasets used here were each originally provided according to different parcellation schemes and different granularity of sampling. To make all data comparable, we opted to use the canonical RM parcellation of the macaque cortex devised by Kötter and Wanke (*55*, *56*, *108*). Tract-tracing and diffusion tractography for this atlas have already been integrated into a high-quality structural connectome (*109*). A human version of this parcellation has likewise been made available, based on functional/cytoarchitectonic landmark-based registration (*57*, *110*). Crucially, the RM atlas was devised with the explicit goal of resolving conflicts between different parcellations in humans and other primate species, by capturing the most consistent cytoarchitectonic, topographic, or functionally defined regions that appear across primate species (*55*, *57*). These considerations make the RM atlas especially well suited for our goal of translating between datasets provided in different parcellations. However, alternative macaque atlases exist (*111*), and cross-species brain mapping is a rapidly evolving field (*7*, *74*, *76*–*78*). Different parcellation approaches (e.g., based on different architectonic features or different imaging modalities) may not always coincide about the exact correspondence between specific cortical locations, especially for densely sampled data such as functional and structural MRI (*7*). Whereas here we have used a common parcellation to compare different data modalities within macaque and between macaque and human while keeping regional identity fixed, future efforts may take the opposite approach. On one hand, combining some of the recently released datasets about different macaque architectural features is bound to yield richer parcellations of the macaque brain (*7*). On the other hand, the availability of gene expression datasets in both humans and macaques may provide the means to enhance existing characterizations of cortical alignment between the two species based on similarity of gene expression, as recently done between human and mouse (*105*).

Nonetheless, despite the limitations that come with needing to map each original dataset onto a common parcellation, we are reassured that this step has not unduly influenced our results. Our findings are highly consistent with the literature in other species (e.g., correspondence of T1w:T2w ratio with myelin-related genes and *PVALB*, *GRIN3A*, and gene PC1; correspondence of gene PC1 across species, which we confirmed with different gene expression datasets for both human and macaque *PVALB*-parvalbumin and *CALB2*-calretining correspondence; and correspondence between *HTR1A* and *5HT*_1A_), suggesting that the quality of our mapping of the different datasets into a common atlas is sufficient to identify a significant correspondence, where one is expected. Note also that we assessed the statistical significance of each correlation against null distributions of maps with preserved spatial autocorrelation, ensuring that our results are not driven by the role of spatial symmetry or granularity of the data (*58*). Last, the RM atlas is the parcellation scheme used by the popular TheVirtualBrain platform for multimodal data sharing and computational modeling (*109*, *112*, *113*), enabling smooth integration of macaque gene and receptor expression data with other available data modalities and brain modeling workflows. In summary, we find moderate within-species gene-receptor correspondence in the macaque cortex, which is improved by taking into account layer specificity. In contrast, there is better interspecies correspondence for many genes underlying fundamental aspects of brain organization, such as cell-type markers, receptor subunits, and myelination. This interspecies correspondence of gene expression exhibits systematic regional heterogeneity, giving rise to a cortical pattern of interspecies divergence that recapitulates the interspecies divergence in cortical morphometry. Throughout, our results demonstrate excellent concordance with findings from the translational neuroscience literature in other organisms (similarity of gene expression between human and mouse; limited gene-receptor correspondence in humans) and also using alternative techniques such as ex vivo immunohistochemistry and in vivo T1w:T2w MRI contrast. Together, the present results showcase both the potential and limitations of macaque spatial transcriptomics as an engine of translational discovery within and across species.

## MATERIALS AND METHODS

### Macaque cortical gene expression from stereo-seq

We used cortex-wide macaque gene expression data recently made available by Chen *et al.* (*16*), who combined single-nucleus RNA-seq (snRNA-seq) with high-resolution, large–field of view spatial transcriptomics from stereo-seq (*36*). Specifically, the authors made available (https://macaque.digital-brain.cn/spatial-omics) postmortem gene expression data covering 143 regions of the left cortical hemisphere of one 6-year-old male cynomolgus macaque (*M. fascicularis*). We refer the reader to the work of Chen *et al.* (*16*) for details. The animal protocol was approved by the Biomedical Research Ethics Committee of CAS Center for Excellence in Brain Science and Intelligence Technology, Chinese Academy of Sciences (ION-2019011). Animal care complied with the guideline of this committee (*16*).

Stereo-seq is a DNA nanoball (DNB) barcoded solid-phase RNA capture method (*36*). It involves reverse transcription of RNAs released from frozen tissue sections fixated onto the stereo-seq chip, and subsequent PCR amplification. The resulting “amplified-barcoded complementary DNA (cDNA) is used as template for library preparation and sequenced” to obtain high-resolution spatially resolved transcriptomics (*36*).

Briefly, Chen *et al.* (*16*) obtained 119 coronal sections at 500-μm spacing, covering the entire cortex of the left hemisphere, which were used for stereo-seq transcriptomics. Adjacent 50-μm-thick sections were also acquired for regional microdissection and snRNA-seq analysis, as well as 10-μm sections adjacent to each stereo-seq section, which were used for the anatomical parcellation of brain regions via immunostaining (*16*). As reported in (*16*), for each coronal section, the cortical region and layer parcellation were manually delineated on stereo-seq data background, based on cytoarchitectural pattern (e.g., cell density and cell size) revealed by total mRNA expression, nucleic acid staining, and NeuN staining of adjacent sections. Each stereo-seq section was stained with a dye specific to nucleic acid and with concanavalin A (ConA). ConA is a fluorescence-labeled lectin that stains the membranes of cells, so that the boundaries between cells are easier to distinguish and manually mark than using nucleic acid staining images alone. On the basis of ConA and nucleic acid staining, the authors then applied an automatic segmentation tool to identify single cells. After automatically registering nucleic acid staining images into mRNA coordinate space based on periodical track lines preengraved in the stereo-seq chip plane, cells in nucleic acid staining images were automatically segmented through a deep learning model trained using annotations by ConA staining. A total of 266,310 segmented cortical cells per section were obtained, with an average of 819 unique molecular identifiers and 458 genes per cell. Although the number of genes captured per cell remains much lower than that obtained by conventional snRNA-seq methods, Chen *et al.* (*16*) note that the average number of genes per cell was much higher than that in background (1.5 ± 0.9 genes per bin), and that this limitation was partially circumvented by using snRNA-seq data to assist cell-type identification (see below). The same authors further confirmed the reproducibility of their results by gene profiles and cell compositions mapped in sections of similar coronal coordinates from three monkeys (*16*).

To obtain gene expression levels, the signal of each gene in all pixels that fell within the segmentation boundaries of the cells was summarized and merged with the location information matrix. The percentage of mitochondrial genes (percent.mt) was calculated with the function “PercentageFeatureSet” (using genes *ND6*, *COX3*, *COX1*, *ND5*, *ND4*, *ND2*, *ND4L*, *ATP8*, *CYTB*, *COX2*, *ND3*, *ATP6*, and *ND1*). Cells with less than 100 features or percent.mt larger than 15% were discarded. Data were then normalized using the function “SCTransform” with the parameter vars.to.regress = “percent.mt.” This step was performed to mitigate potential batch effects resulting from variations in single-cell sequencing depth within and across sections of the stereo-seq map. These normalized gene expression data are made available at https://macaque.digital-brain.cn/spatial-omics for 143 cortical regions of the left hemisphere, including prefrontal, frontal, cingulate, somatosensory, insular, auditory, temporal, parietal, occipital, and piriform areas. On the data-sharing portal, separate normalized gene expression data are made available for each region and for each of its cortical layers. Except for the layer-specific analyses, we use the region-level aggregated data. To make the gene expression comparable across our datasets, the combined gene expression across layers was manually mapped onto the cortical regions of the RM macaque atlas of Kötter and Wanke (*55*), mirroring data between hemispheres.

Chen *et al.* (*16*) also performed cell-type classification using snRNA-seq data for 1,493,240 cells from 143 regions of the entire cerebral cortex of two macaques. Dimensionality reduction and clustering of this snRNA-seq dataset led to the identification of 264 cell clusters, which were further grouped into 23 cell subclasses belonging to the three well-known classes of brain cells: glutamatergic neurons (with 10 subclasses), GABA-ergic neurons (with 7 subclasses), and nonneuronal cells (6 subclasses). The 10 glutamatergic neuron subclasses were annotated by their layer preferences (L for layer: L2, L2/3, L2/3/4, L3/4, L3/4/5, L4, L4/5, L4/5/6, L5/6, and L6). The seven GABAergic neuron subclasses were divided into chandelier cells (CHC) and cells preferentially expressing lysosome-associated membrane protein 5 (labeled LAMP5), vasoactive intestinal peptide (VIP), reelin (RELN), VIP and reelin (VIP-RELN), parvalbumin (PV), and somatostatin (SST). The six nonneuronal subclasses included astrocytes, oligodendrocyte precursor cells, oligodendrocytes, microglia, endothelial cells, and VLMCs (*16*). The molecular fingerprints of classified cell types from snRNA-seq were then used to register and annotate spatially resolved single cells in stereo-seq spatial transcriptome maps, using a recently developed cell-type annotation algorithm, Spatial-ID, which combines existing knowledge of reference single-cell RNA-seq data with spatial information of spatially resolved transcriptomics data (*16*). For each cell type, the authors quantified the density in each layer and cortical region by dividing the total number of cells in each area by the area size. The robustness of cell annotation was evidenced by high correlation value between the gene expression profiles of annotated cell types and those of snRNA-seq–defined cell types (*16*). The resulting regional density of each cell type is also made available at https://macaque.digital-brain.cn/spatial-omics.

### Macaque receptor density from in vitro receptor autoradiography

In vitro autoradiography data for 14 neurotransmitter receptors were obtained from (*40*): *AMPA*, *kainate*, *NMDA*, *GABA*_A_, *GABA*_B_, *GABA*_A/BZ_, *M*_1_, *M*_2_, _3_, α_1_, α_2_, *5HT*_1A_, *5HT*_2A_, and *D*_1_. The authors applied quantitative in vitro receptor autoradiography to label 14 neurotransmitter receptors in three male *M. fascicularis* brains (7.3 ± 0.6 years old; body weight 6 ± 0.8 kg) obtained from Covance Preclinical Services, where they were housed and used as control animals for pharmaceutical studies performed in compliance with legal requirements. Animal experimental procedures and husbandry had the approval of the respective Institutional Animal Care and Use Committee and were carried out in accordance with the European Council Directive of 2010 (*40*). We refer the reader to the work of Froudist-Walsh *et al.* (*40*) for details.

Briefly, brain tissue was serially sectioned in the coronal plane (section thickness, 20 μm) using a cryostat microtome (Leica, CM3050S). Sections were thaw mounted on gelatine-coated slides, sorted into 22 parallel series and freeze dried overnight. Receptor binding protocols encompass a preincubation to rehydrate sections, a main incubation with a tritiated ligand in the presence or absence of a nonlabeled displacer and a final rinsing step to terminate binding. Incubation with the tritiated ligand alone demonstrates total binding; incubation in combination with the displacer reveals the proportion of nonspecific binding sites. Specific binding is the difference between total and nonspecific binding and was less than 5% of the total binding. Thus, total binding is considered to be equivalent of specific binding. Sections were exposed together with standards of known radioactivity against tritium-sensitive films (Hyperfilm, Amersham) for 4 to 18 weeks depending on the receptor type. Ensuing autoradiographs were processed by densitometry with a video-based image analyzing technique. In short, autoradiographs were digitized as 8-bit images. Gray values in the images of the standards were used to compute a calibration curve indicating the relationship between gray values in an autoradiograph and binding site concentrations in femtomole per milligram (fmol mg^−1^) of protein. Concentrations of radioactivity (R, counts per minute) in each standard, which had been calibrated against brain tissue homogenate, were converted to binding site concentrations (Cb, fmol mg^−1^ of protein). The ensuing calibration curve was used to linearize the autoradiographs—that is, to convert the gray value of each pixel into a binding site concentration in fmol mg^−1^ of protein (*40*).

The data of density of receptors per neuron were made available for 109 cortical areas of the macaque brain, which were identified based on their cytoarchitecture and receptor-architecture characteristics (*40*). To enable comparison across datasets, we resampled these data to the RM macaque cortical parcellation of Kötter and Wanke (*55*). The macaque receptor data were manually mapped from the original macaque Yerkes19 to the macaque D99 atlas in NMT (National Institute of Mental Health Macaque Template) v1.3 space (*114*). The RheMap toolbox (https://github.com/PRIME-RE/RheMAP) was used to bring the RM atlas in NMT 1.3 macaque space. Last, once both the RM atlas and the receptor maps were in the same NMT space, the RM atlas was used to parcellate each receptor map (see fig. S30, A and B, for examples of the resulting mapping and corresponding gene expression maps).

For visual area V1 and six subregions of the inferior parietal lobe (PF, PFG, PG, Opt, PGop, and PFop), layer-specific receptor density was also obtained. Equidistant receptor profiles oriented perpendicular to the cortical surface were extracted from the digitized autoradiographs to quantify receptor density across the cortical depth. To account for variations in cortical thickness, the length of each profile was normalized using linear interpolation to a uniform cortical thickness of 100%. By comparing the receptor autoradiographs with adjacent cell body–stained sections, the position and thickness of each cortical layer could be determined along the entire cortical depth. Consequently, the receptor profiles were divided into segments corresponding to these layers. The area beneath each segment of the receptor profile was used to calculate the receptor density for each identified layer (*64*, *115*). Because layer-specific data for these regions were available for both macaque gene expression and macaque receptor density, mapping to the RM atlas was not required.

### Human gene expression

Regional human gene expression profiles were obtained using microarray data from the AHBA (*15*), with preprocessing as recently described (*116*). The AHBA is a publicly available transcriptional atlas containing gene expression data measured with DNA microarrays and sampled from hundreds of histologically validated neuroanatomical structures across normal postmortem human brains from six donors (five male and one female; age, 24–55 years). We extracted and mapped gene expression data to the 82 cortical regions of interest (ROIs) of our parcellation using the abagen toolbox [https://abagen.readthedocs.io/ (*39*)]. Data were pooled between homologous cortical regions to ensure adequate coverage of both left (data from six donors) and right hemisphere (data from two donors). Distances between samples were evaluated on the cortical surface with a 2-mm distance threshold. Only probes where expression measures were above a background threshold in more than 50% of samples were selected. A representative probe for a gene was selected based on the highest intensity. Gene expression data were normalized across the cortex using scaled, outlier-robust sigmoid normalization. A total of 15,633 genes survived these preprocessing and quality assurance steps. For comparison with the macaque data, the human gene expression data were parcellated into a human-adapted version of the cortical parcellation of Kötter and Wanke (*55*), as adapted to the human brain by Bezgin *et al.* (*57*) (see fig. S30, A and B). We only included in our analyses genes with a one-to-one ortholog between *H. sapiens* and *M. fascicularis*. We also replicate our main results using human gene expression data from an alternative modality, RNA-seq, which was available from two of six AHBA donors (*15*).

### Gene-receptor pairs

Neurotransmitter receptors are protein complexes bound in the cell membrane. They can be classified as ionotropic (if signal transduction is mediated by ion channels) or metabotropic (G protein coupled). Ionotropic receptors are multimeric complexes consisting of multiple subunits, each encoded by a distinct gene. Metabotropic receptors are monomeric complexes: Although there are several associated second messenger components, there is only a single protein embedded in the membrane, which is therefore encoded by a single gene. Therefore, for monomeric receptors, we correlated protein density with expression of the corresponding gene. For multimeric receptors, which involve distinct subunits, in our main analysis, we correlated protein density with expression of genes coding for the same subunits as used in (*47*).

1. *GABA*_A_: This pentamer can be coded by up to 19 subunits. In our main analysis, we show correlations with genes coding for the three primary subunits: α_1_, β_2_ and γ_2_.

2. *GABA*_B_: Our main analysis reports correspondence with genes coding for both subunits.

3. *AMPA*: In our main analysis, we show correlations with the *GRIA1* gene. Correlations with additional genes as used in (*48*) are reported in the Supplementary Materials.

4. *NMDA*: In our main analysis, we show correlations with the *GRIN1* gene, which encodes the N1 subunit. Correlations with additional genes as used in (*48*) are reported in the Supplementary Materials.

5. *Kainate*: We show results for the *GRIK2* gene in our main analysis. Correlations with additional genes as used in (*48*) are reported in the Supplementary Materials.

Because macaque gene expression data from (*16*) do not include the *CHRM3* gene that codes for the *M*_3_ receptor, this receptor could not be included in our main gene-receptor comparison. Macaque gene expression was also not available for *ADRA1C* (pertaining to the α_1_ receptor), so we only included genes *ADRA1A* and *ADRA1B*. Likewise, *ADRA2B* and *ADRA2C* genes (pertaining to the α_2_ receptor) were not available, so we only used *ADRA2A*.

### Macaque T1w:T2w map, dendritic spines, and parvalbumin and calretinin immunohistochemistry

For our main analysis, we used the map of in vivo macaque intracortical myelination from T1w:T2w ratio, originally from (*117*) and made available by Froudist-Walsh *et al.* (*40*). These data were available in the same 109-area parcellation of the macaque cortex as the receptor data. We therefore followed the same procedure and resampled these data to the RM macaque cortical parcellation of Kötter and Wanke (*55*).

Burt *et al.* (*42*) assembled data on the immunohistochemically measured densities of calretinin (also known as calbindin-2) and parvalbumin-expressing inhibitory interneurons for several macaque brain areas, from multiple immunohistochemistry studies (*65*–*68*). The same authors also provide T1w:T2w intracortical myelination data for the same regions (*42*), which we used for our replication analysis. Last, Burt *et al.* (*42*) also provide data about the number of spines of basal-dendritic trees of layer 3 pyramidal neurons. To compare data across modalities, we manually mapped their data onto 38 bilateral regions of the RM parcellation (*55*).

In addition to the aggregated data of Burt *et al.* (*42*), we also used immunohistochemistry data about the prevalence of parvalbumin-immunoreactive and calretinin-immunoreactive neurons for a subset of macaque visual, auditory, and somatosensory cortical regions from (*68*), which was one of the original studies combined by Burt *et al.* (*42*). Data were obtained from three normal adult macaque monkeys (*Macaca fuscata*), with approval from the animal research committee of RIKEN (Japan) and in accordance with the Guiding Principles for the Care and Use of Animals in the Field of Physiological Sciences of the Japanese Physiological Society (*68*). Briefly, a monoclonal antiparvalbumin antibody (Sigma) and a polyclonal anticalretinin antiserum (SWant) were used on postmortem brain sections (30 μm of thickness). The number of parvalbumin-immunoreactive and calretinin-immunoreactive neurons (all nonpyramidal except for a few calretinin-immunoreactive pyramidal neurons in area 28) was counted in 200-μm-wide strips extending vertically to the cortical surface through all layers, and for each cortical area, this process was repeated 16 times at different locations in the three monkeys (*68*). Here, we ranked areas based on their mean count of immunoreactive neurons (separately for calretinin and parvalbumin) as displayed in figure 4 of (*68*) and subsequently manually mapped these ranks onto bilateral regions of the RM parcellation (*55*).

### Brain-related genes

In addition to the lists of receptor-related genes from (*47*, *48*), we also obtained from Fulcher *et al*. (*44*) a list of 124 brain-related genes coding for neurotransmitter and neuropeptide receptors and subunits, as well as cell-type markers for parvalbumin, somatostatin, vasoactive intestinal peptide, calbindin, and the four most abundant genes in myelin. Of these, 99 genes were available and passed our quality control in both macaque and human, and were included in our analysis.

### Alternative macaque gene expression data from RNA-seq

Bo *et al.* (*17*) made available bulk RNA-seq regional expression patterns of 18 genes pertaining to neurotransmitter receptors, transporters, and synthesis from the brain of *M. fascicularis*. Expression patterns are provided for 97 regions of the D99 macaque atlas, and we manually mapped them onto the RM parcellation (*55*). As reported in the original study (*17*), nine adult cynomolgus monkeys (*M. fascicularis*; mean ± SD, 13.6 ± 7.8 years, eight males and one female) weighing 4.2 to 12.0 kg (8.6 ± 2.6 kg) were used for the study. All animal experimental procedures were approved by the Animal Care and Use Committee of CAS Center for Excellence in Brain Science and Intelligence Technology, Chinese Academy of Sciences, and conformed to National Institutes of Health guidelines for the humane care and use of laboratory animals. We refer the reader to the original study (*17*) for details of regional bulk RNA-seq procedures.

For the comparison between macaque RNA-seq gene expression and receptors, because only a small number of macaque genes are available from (*17*), we compare these genes against all available macaque receptors pertaining to the same neurotransmitter systems. In addition to GABA and glutamate receptors, we also include in the comparison all available cholinergic receptors (*M*_1_, *M*_2_, and *M*_3_) against both acetylcholine-related genes in the RNA-seq dataset: *CHAT* (acetylcholine synthesis) and *CHRNA1* (nicotinic receptor). Likewise, we compare both α_1_ and α_2_ adrenergic receptors against *ADRA2C* (α_2_ receptor) and *PNMT* (epinephrine synthesis); we compare both serotonin receptors (*5HT*_1A_ and *5HT*_2A_) with both available serotonin-related genes, *HTR1B* and *HTR2C*, and the only available dopamine receptor (*D*_1_) against all dopamine-related genes in the RNA-seq dataset: *DRD2* (*D*_2_ receptor), *SLC6A2* (dopamine reuptake), *NTS* (dopamine breakdown), and *TH* (dopamine synthesis).

### Macaque anatomical connectivity

Anatomical connectivity for the macaque brain was obtained from the fully weighted, whole-cortex macaque connectome recently developed by Shen *et al.* (*109*). This connectome was generated by combining information from two different axonal tract-tracing studies from the CoCoMac database (http://cocomac.g-node.org/main/index.php) (*118*) with diffusion-based tractography obtained from nine adult macaques (*Macaca mulatta* and *M. fascicularis*) (*109*). The resulting connectome provides a matrix of weighted, directed anatomical connectivity between each of the cortical ROIs of the RM atlas of Kötter and Wanke (*109*).

### Macaque-human cortical expansion

To contextualize our regional pattern of interspecies correlation of gene expression, we used neuromaps (https://netneurolab.github.io/neuromaps/) (*119*) to obtain the map of cortical expansion between macaque and human from (*77*), parcellated into Schaefer-400 cortical atlas.

### Statistical analyses

Correspondence across cortical regions was quantified using Spearman’s rank-based correlation coefficient, which is more robust to outliers and nonnormality than Pearson correlation and is recommended when studying the correlation between mRNA and protein levels. To control for the spatial autocorrelation inherent in neuroimaging data, which can induce an inflated rate of false positives (*58*), we assessed the statistical significance of correlations nonparametrically, by comparing each empirical correlation against a distribution of 10,000 correlations with null maps having the same spatial autocorrelation. This approach embodies the null hypothesis that the empirically observed correlation is simply due to the spatial autocorrelation in the data, such that a similar correlation would be observed with random maps, so long as the spatial autocorrelation is the same. Null maps with preserved spatial autocorrelation were generated using Moran Spectral Randomization on the inverse Euclidean distances between parcel centroids, as implemented in the BrainSpace toolbox (https://brainspace.readthedocs.io/en/latest/) (*59*). Moran Spectral Randomization quantifies the spatial autocorrelation in the data in terms of Moran’s *I* coefficient (*120*), by computing spatial eigenvectors known as Moran eigenvector maps. The Moran eigenvectors are then used to generate null maps by imposing the spatial structure of the empirical data on randomized surrogate data (*58*, *59*). The resulting null maps are therefore random, but with the same level of spatial autocorrelation as the empirical data. If a more extreme level of correlation is consistently observed between the empirical brain maps than with the null maps, then we can conclude that this association is not simply due to spatial autocorrelation.

For the correlations with multimeric receptors, the regional distribution of the same receptor is being compared against multiple genes (each coding for a different subunit of the receptor); therefore, in addition to showing the individual *P* values in the main text, in the Supplementary Materials, we also show results after applying the FDR correction for multiple comparison. Likewise, in the Supplementary Materials, we show results after applying FDR correction when correlating the same receptor against multiple layer-specific expression patterns of the same gene.

To evaluate the potential role of long-range axonal projections in mediating gene-receptor correlations, we generate an updated regional map for each gene, as follows

$${x\prime }_{i}=\frac{\sum_{j\in 𝒩\left(i\right)}\;w_{\mathrm{ij}}\;x_{j}}{\sum_{j\in 𝒩\left(i\right)}\;w_{\mathrm{ij}}}$$

where ${x\prime }_{i}$ is the updated gene expression level for region i; $x_{j}$ is the original gene expression level of region j, which region i is connected to; $w_{\mathrm{ij}}$ is the weight of the structural connection from region i to region j, representing the strength of the connection; and 𝒩(i) is the set of regions that region i has outgoing structural connections to.

In other words, for each region, we take a weighted average of the gene expression levels of the regions that it has outgoing structural connections to, where the weight is given by the strength of the connection (*34*). Comparisons between proportions of significantly correlated patterns between different neurotransmitter types were performed using the χ^2^ test.
