# Supplementary material for: Benchmarking macaque brain gene expression for horizontal and vertical translation
Source: Sci Adv. 2025 Feb 28;11(9):eads6967. doi: 10.1126/sciadv.ads6967 (PMC11870082; doi:10.1126/sciadv.ads6967)
Supplement: Supplementary file 1 — Figs. S1 to S30 [file sciadv.ads6967_sm.pdf]

Science Advances
AAAS

Supplementary Materials for

Benchmarking macaque brain gene expression for horizontal and vertical translation

Andrea I. Luppi *et al.*

Corresponding author: Andrea I. Luppi, andrea.luppi@psych.ox.ac.uk

Sci. Adv. **11**, eads6967 (2025)
DOI: 10.1126/sciadv.ads6967

This PDF file includes:

Figs. S1 to S30

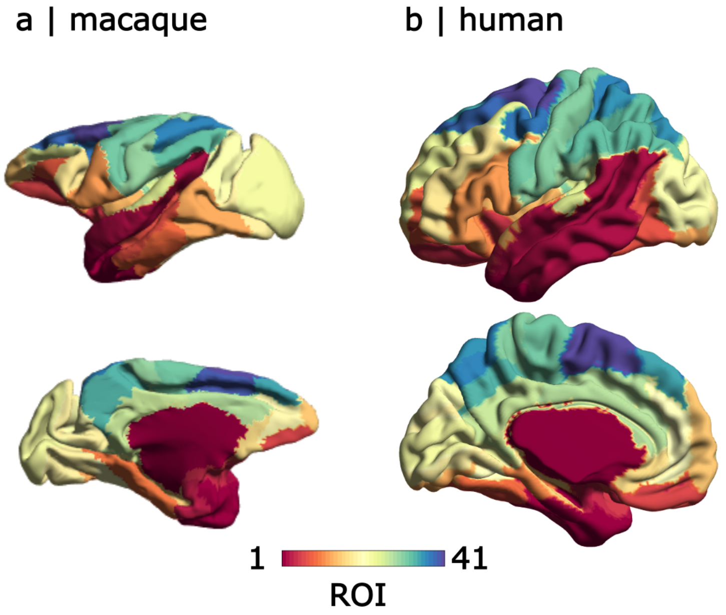

Figure S1. **Regional Mapping parcellation for different species |** (**a**) An original depiction of the macaque cortical atlas developed by Kötter and Wanke (55), shown on the macaque cortical surface. (**b**) An original depiction of the human translation of the same atlas, introduced by (57).

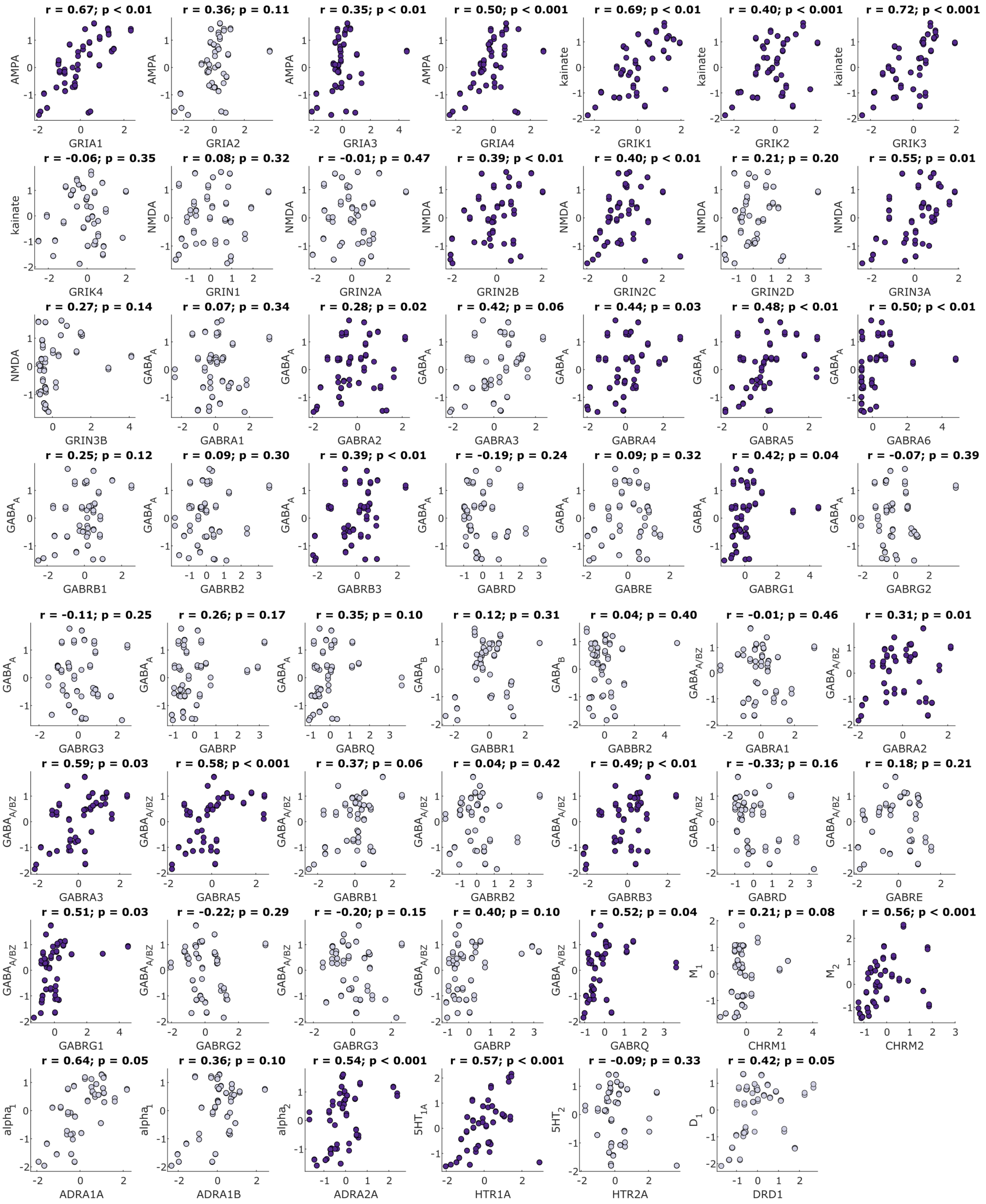

Figure S2. **Macaque cortical receptor densities versus gene expression in the macaque, for all pairs |** Indigo scatter plots indicate significant human-macaque correspondence (Spearman's r, $p < 0.05$ against a null distribution of surrogate cortical maps with preserved spatial autocorrelation). Values are z-scored.

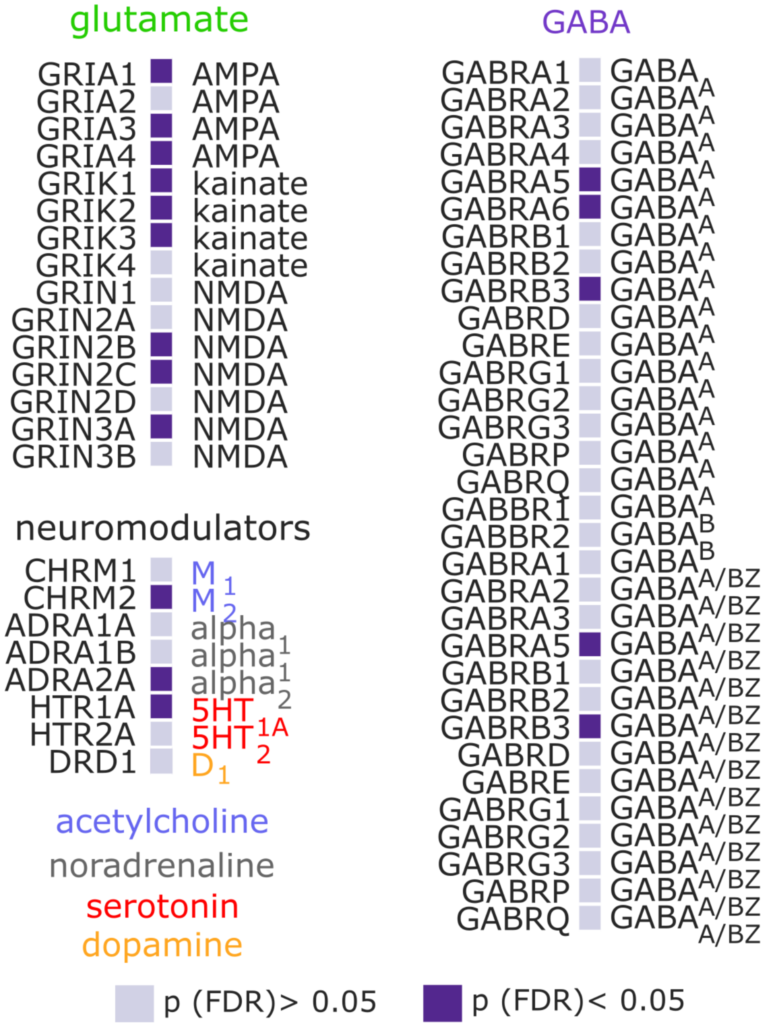

Figure S3. **Significance of correlations between macaque cortical receptor density and gene expression, adjusted for multiple comparisons |** Indigo cells indicate significant correlation across regions (Spearman's r, $p < 0.05$ against a null distribution of surrogate cortical maps with preserved spatial autocorrelation), after applying FDR correction for multiple comparisons across all genes matched with the same receptor.

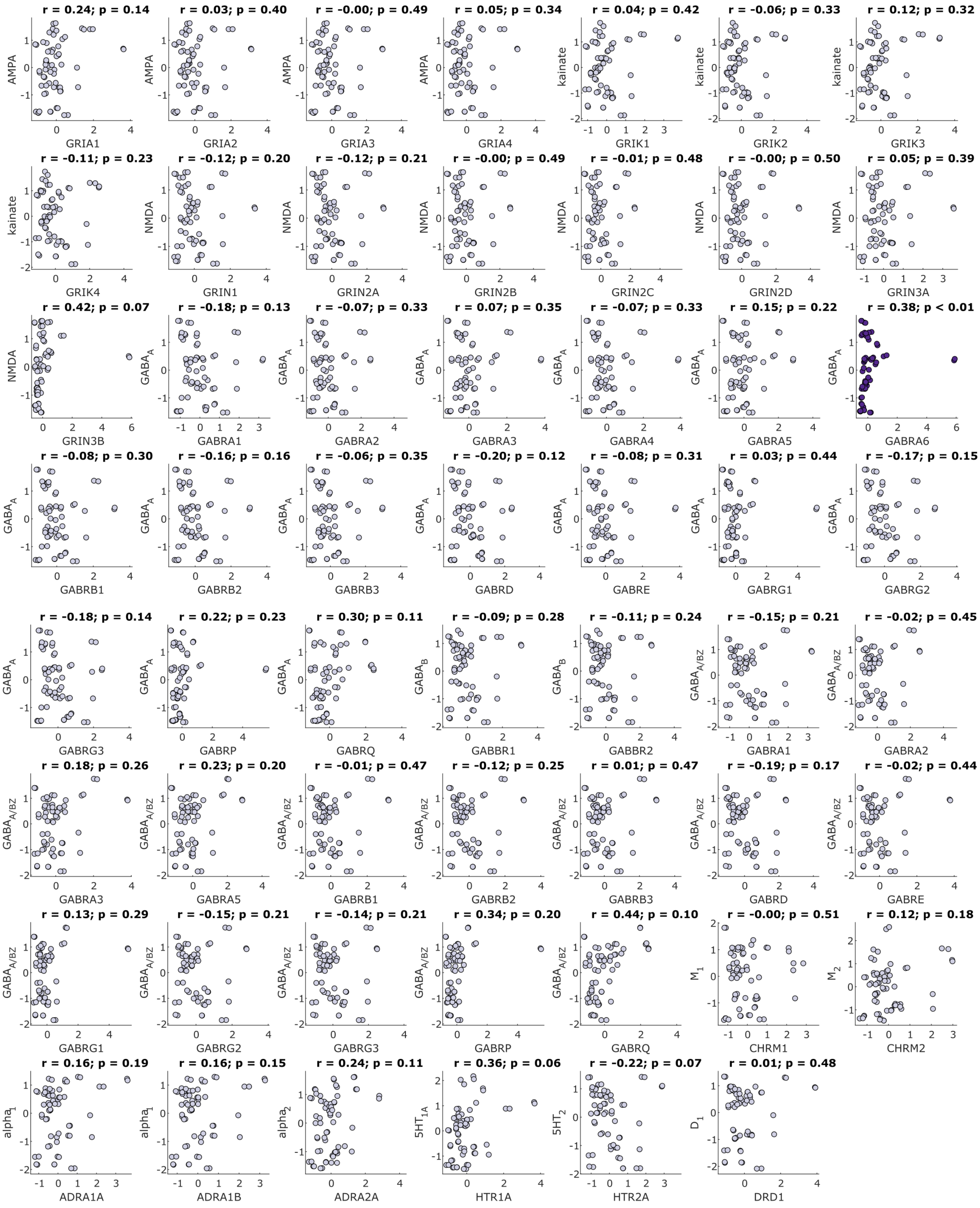

Figure S4. **Macaque cortical receptor densities versus mean gene expression of each region's neighbours, weighted by efferent anatomical connection strength |** Indigo scatter plots indicate significant human-macaque correspondence (Spearman's *r*, $p < 0.05$ against a null distribution of surrogate cortical maps with preserved spatial autocorrelation). Values are z-scored.

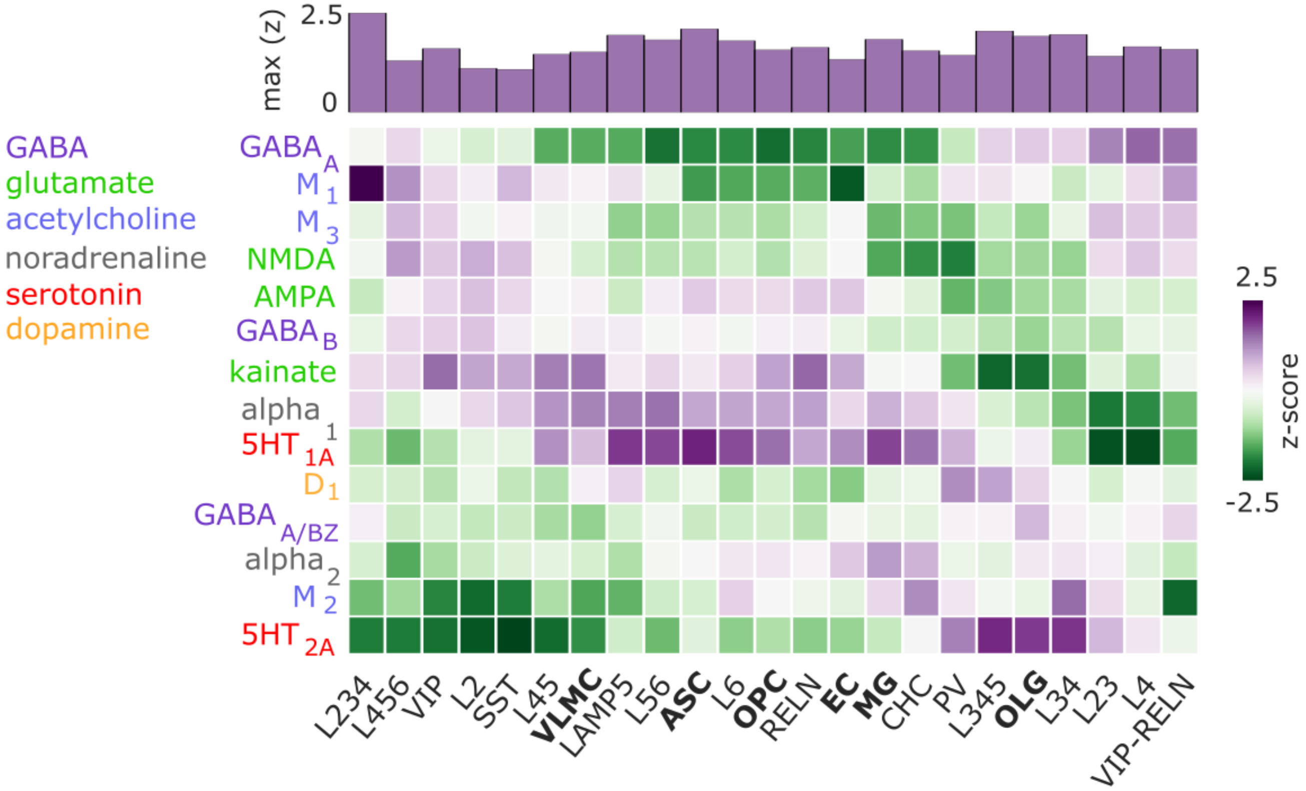

Figure S5. **Preferential association of transcriptomically-derived macaque cell classes for different receptors |** Data are z-scored column-wise, so a high value indicates that a specific cell type has above-average association with the corresponding receptor, compared with other receptors. Histogram at the top indicates the maximum z-score observed for each cell type, reflecting relative specificity of association. As per the naming convention from (16), glutamatergic neuron subclasses were annotated by their layer preferences (L for layer: L2, L2/3, L2/3/4, L3/4, L3/4/5, L4, L4/5, L4/5/6, L5/6, and L6). GABAergic neuron subclasses were divided into cells preferentially expressing lysosome-associated membrane protein 5 (LAMP5), vasoactive intestinal peptide (VIP), reelin (RELN), VIP and reelin (VIP-RELN), parvalbumin (PV), somatostatin (SST), and chandelier cells (CHC). The 6 non-neuronal subclasses included astrocytes (ASC), oligodendrocyte precursor cells (OPC), oligodendrocytes (OLG), microglia (MG), endothelial cells (EC), and vascular leptomeningeal cells (VLMC). Cell types pertaining to non-neuronal cells are shown in bold to highlight their association with serotonin receptors. Bars at the top indicate the maximum z-score for each cell type.

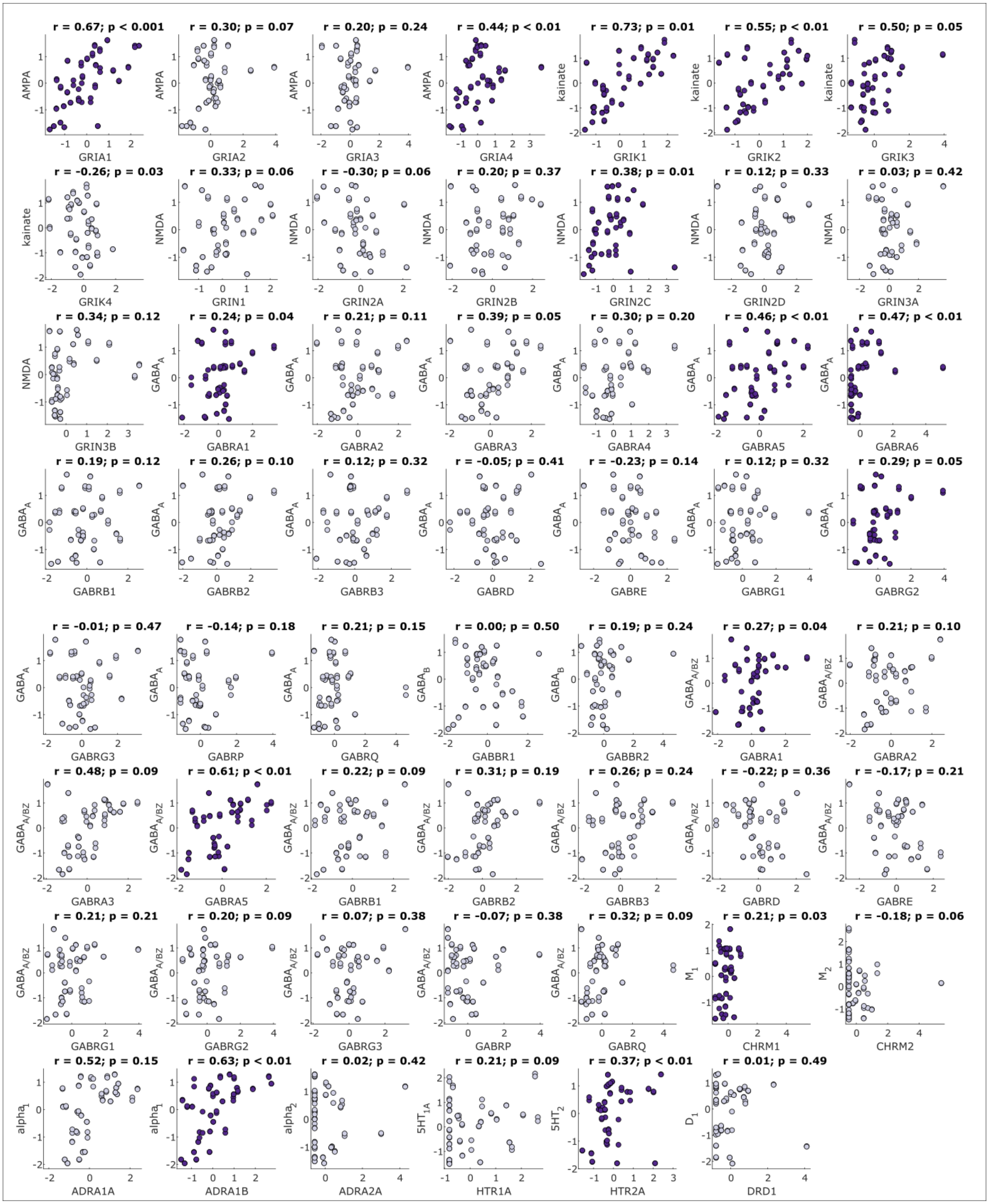

Figure S6. **Cortical receptor densities versus Layer 1 gene expression in the macaque, for all pairs |** Indigo scatter plots indicate significant human-macaque correspondence (Spearman's r, $p < 0.05$ against a null distribution of surrogate cortical maps with preserved spatial autocorrelation). Values are z-scored.

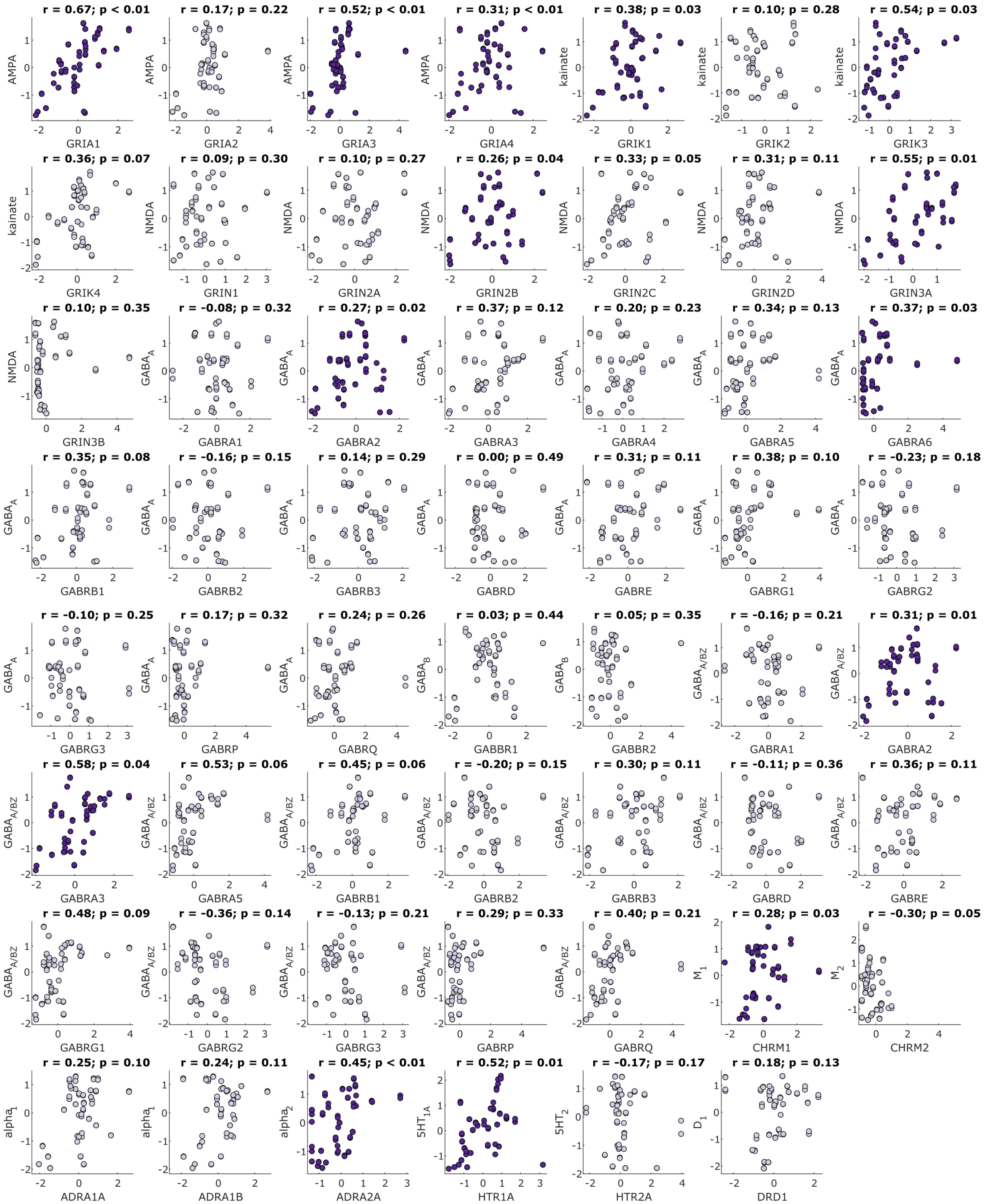

Figure S7. **Cortical receptor densities versus Layer 2 gene expression in the macaque, for all pairs |** Indigo scatter plots indicate significant human-macaque correspondence (Spearman's r, $p < 0.05$ against a null distribution of surrogate cortical maps with preserved spatial autocorrelation). Values are z-scored.

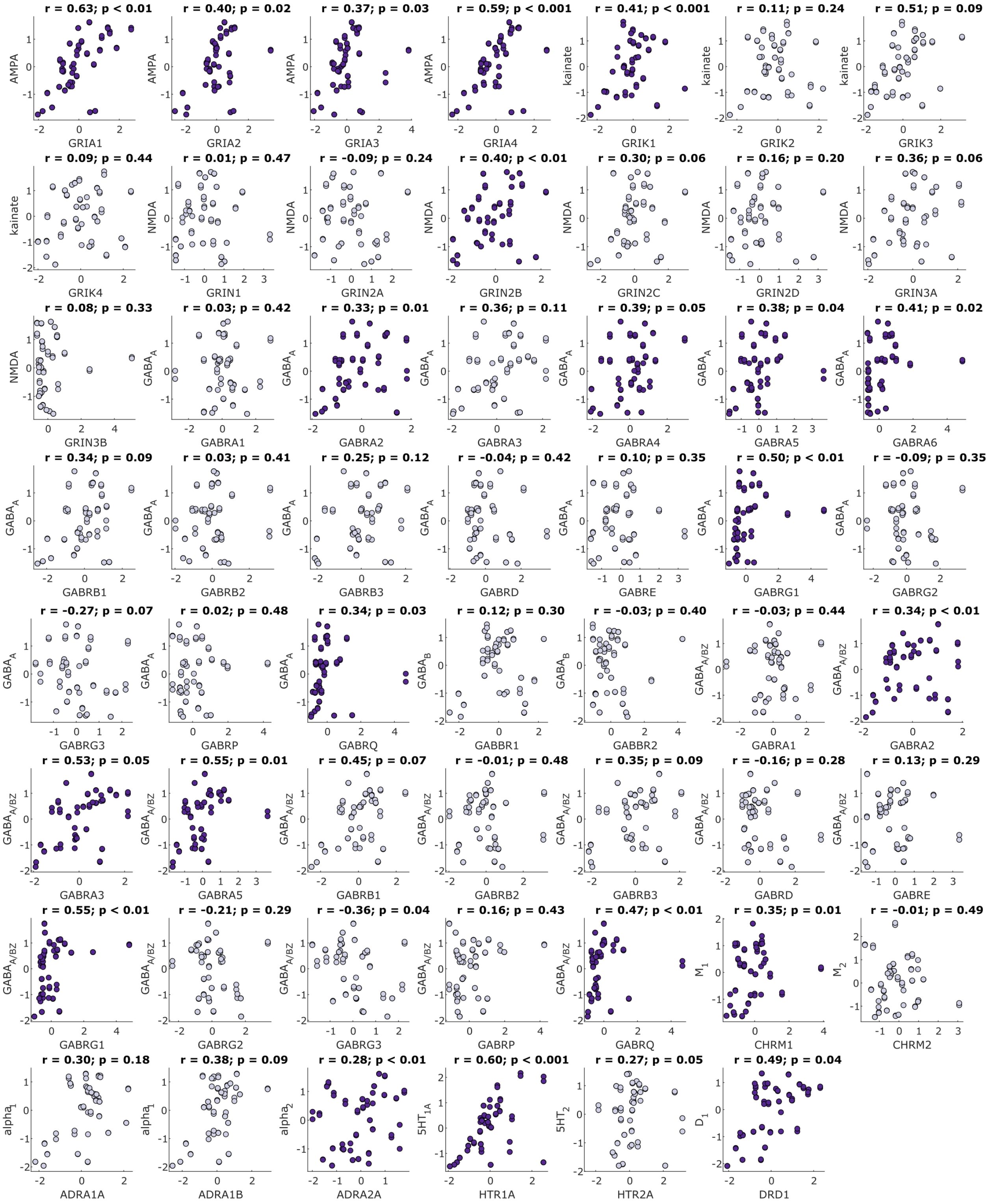

Figure S8. **Cortical receptor densities versus Layer 3 gene expression in the macaque, for all pairs |** Indigo scatter plots indicate significant human-macaque correspondence (Spearman's r, $p < 0.05$ against a null distribution of surrogate cortical maps with preserved spatial autocorrelation). Values are z-scored.

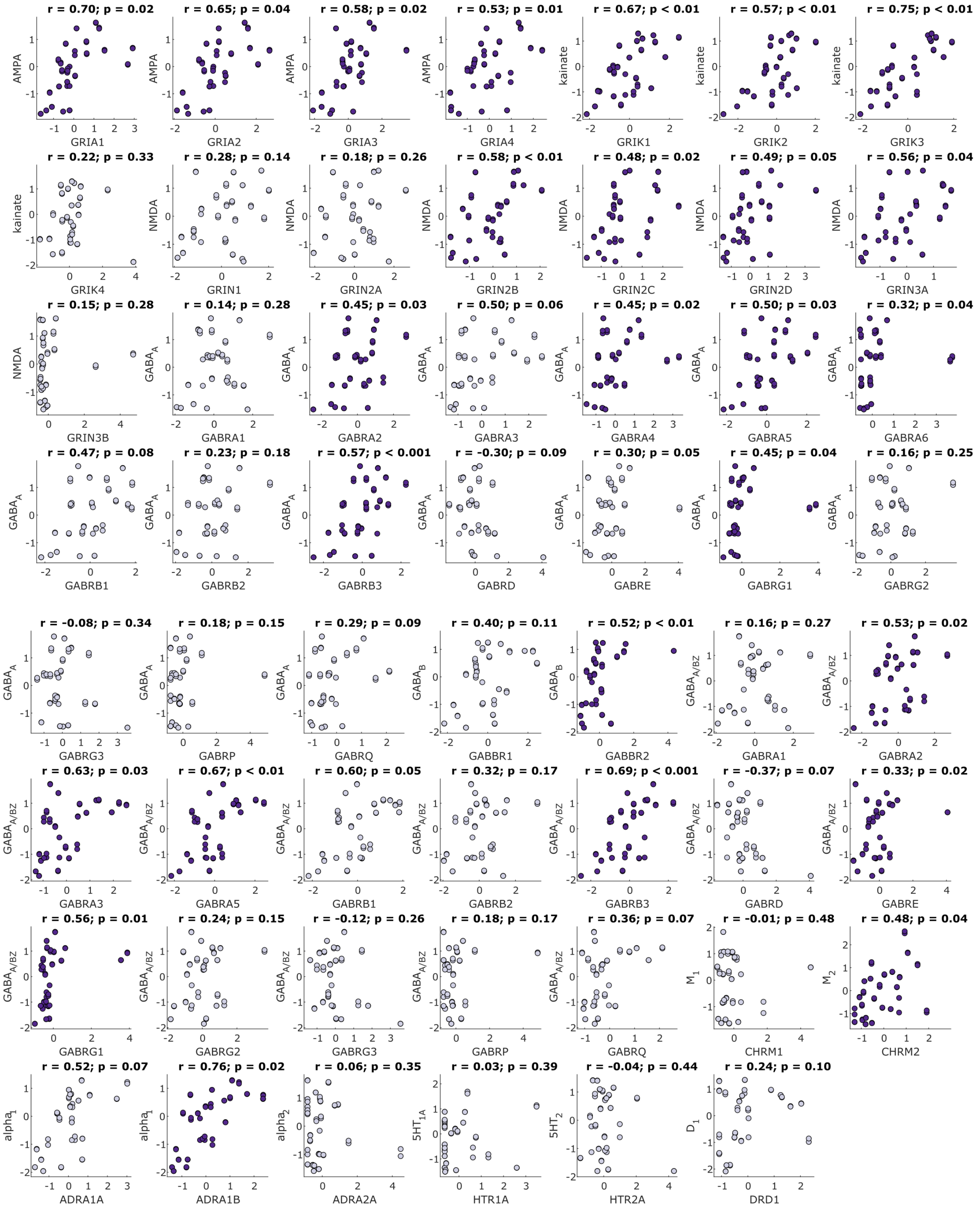

Figure S9. **Cortical receptor densities versus Layer 4 gene expression in the macaque, for all pairs |** Indigo scatter plots indicate significant human-macaque correspondence (Spearman's r, $p < 0.05$ against a null distribution of surrogate cortical maps with preserved spatial autocorrelation). Values are z-scored.

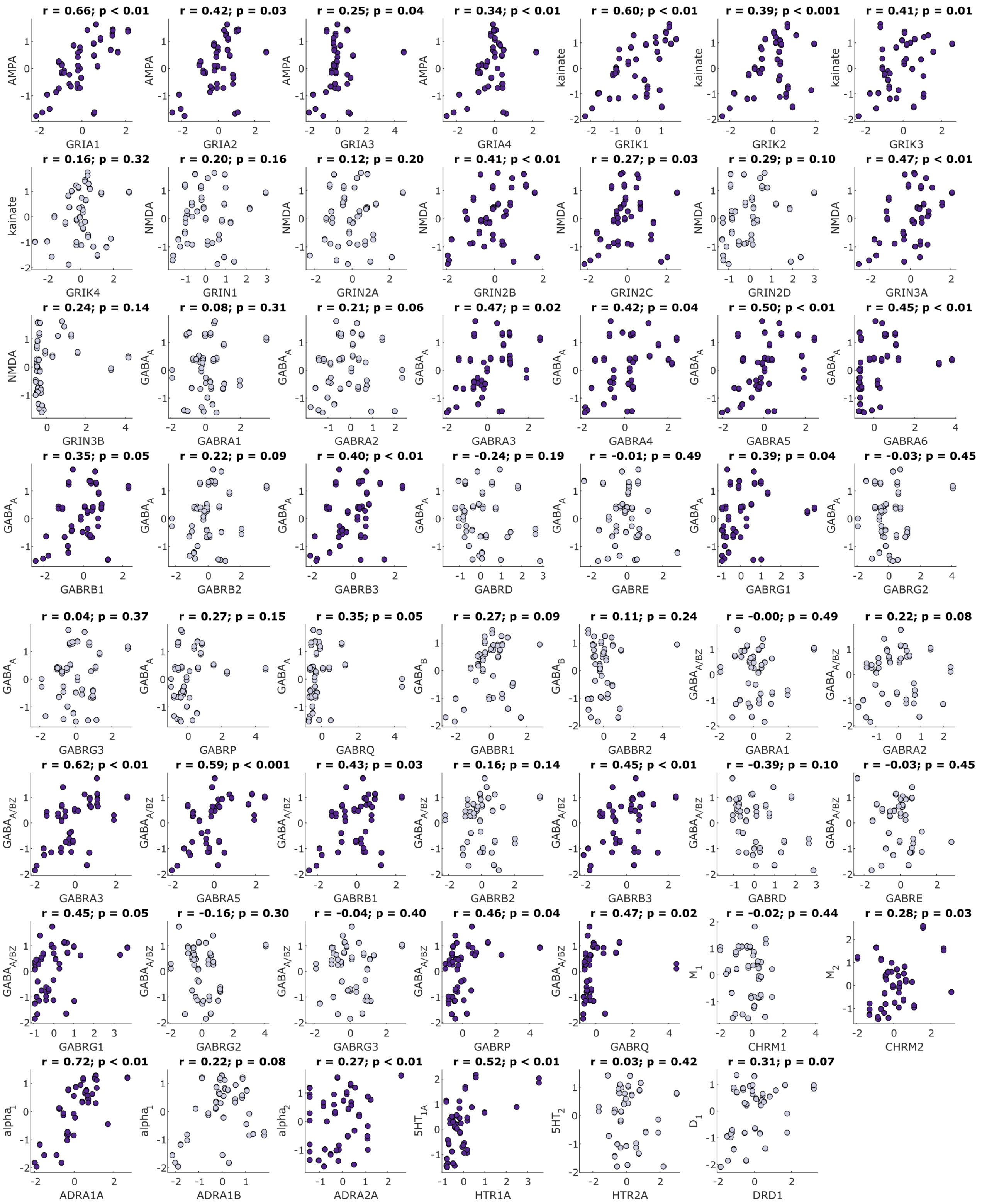

Figure S10. **Cortical receptor densities versus Layer 5 gene expression in the macaque, for all pairs |** Indigo scatter plots indicate significant human-macaque correspondence (Spearman's r, $p < 0.05$ against a null distribution of surrogate cortical maps with preserved spatial autocorrelation). Values are z-scored.

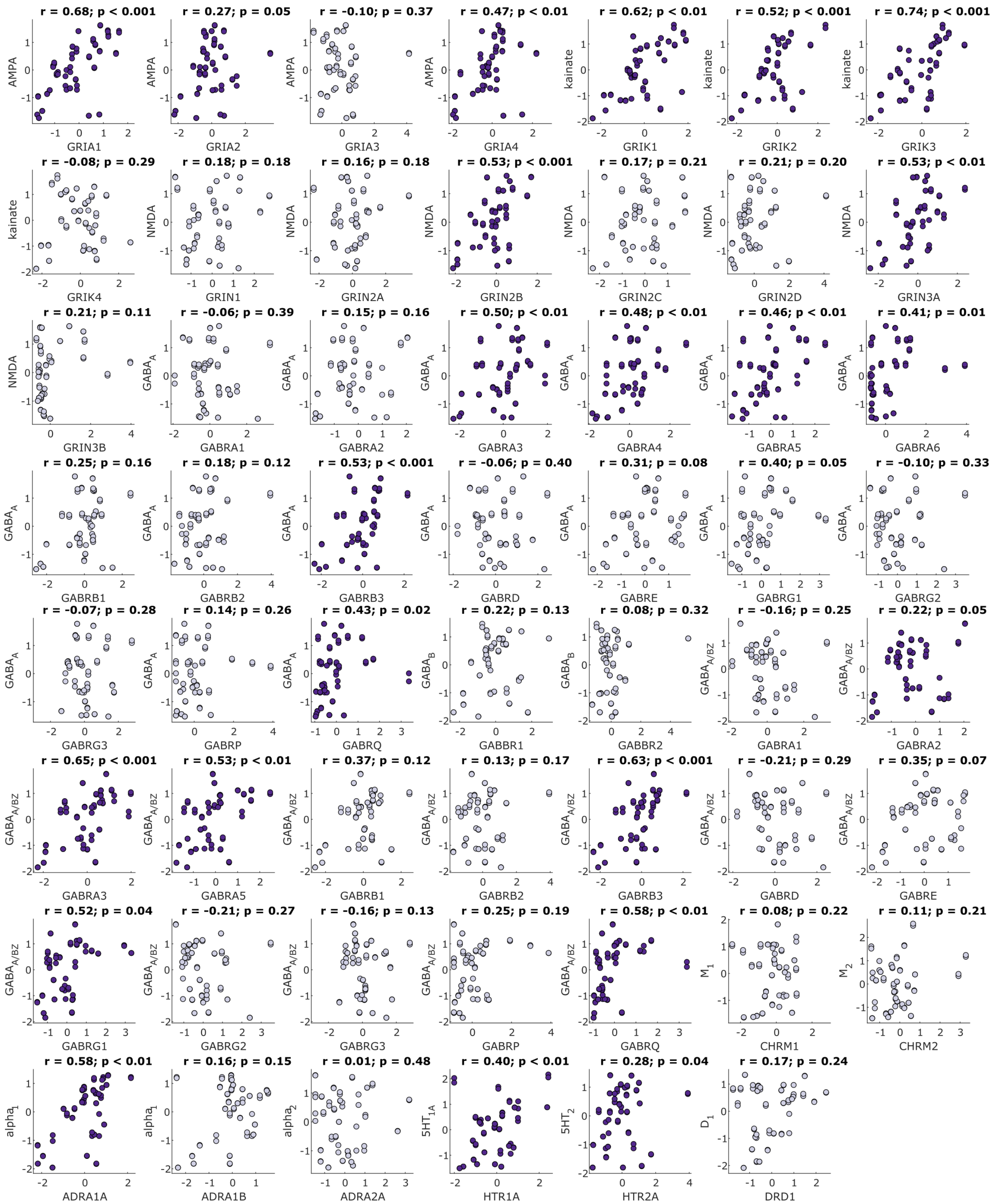

Figure S11. **Cortical receptor densities versus Layer 6 gene expression in the macaque, for all pairs |** Indigo scatter plots indicate significant human-macaque correspondence (Spearman's r, p < 0.05 against a null distribution of surrogate cortical maps with preserved spatial autocorrelation). Values are z-scored.

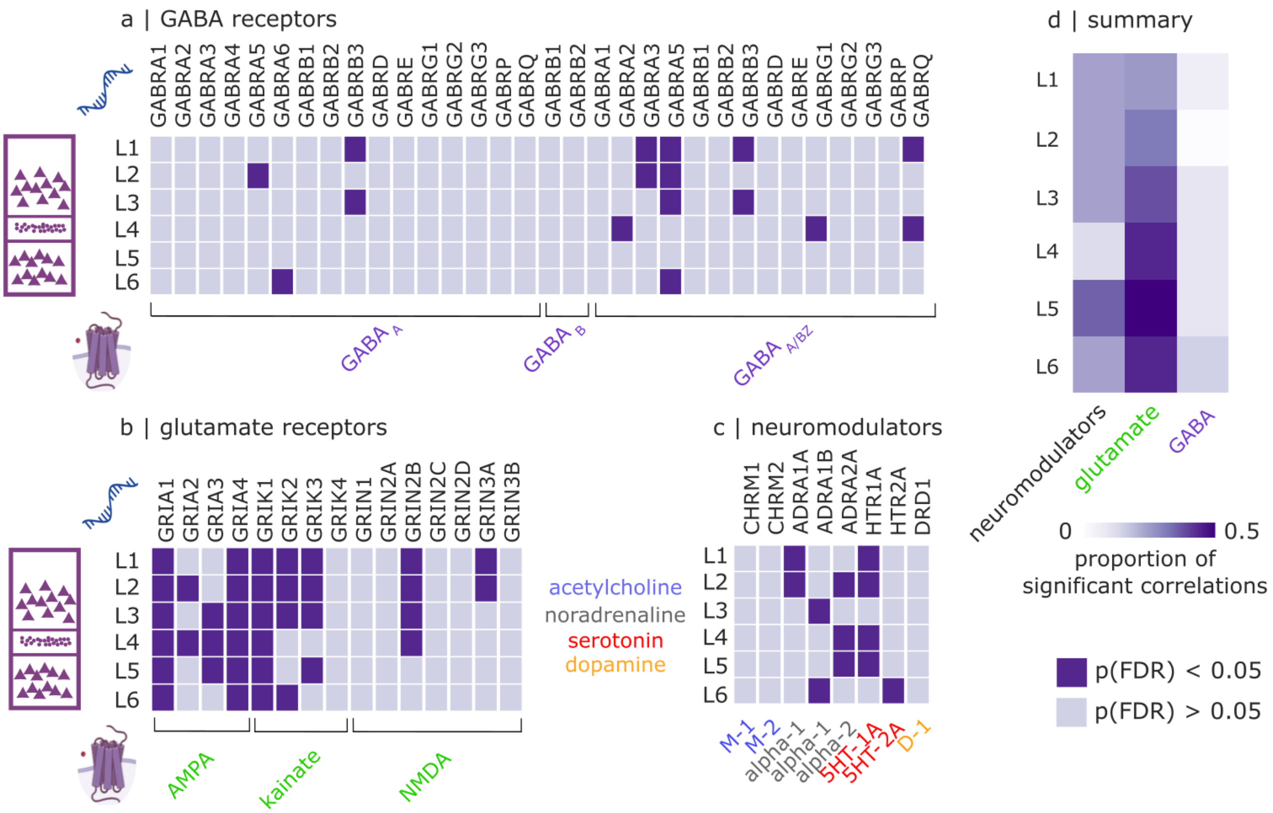

Figure S12. **Correspondence between macaque cortical gene expression, and macaque cortical receptor expression across cortical layers and receptor types, adjusted for multiple comparisons |** (**a**) Significance of gene-receptor correlations for *GABA* receptors. (**b**) Significance of gene-receptor correlations for glutamate receptors. (**c**) Significance of gene-receptor correlations for receptors pertaining to neuromodulatory systems (acetylcholine, noradrenaline, serotonin, dopamine). For a-c, indigo cells indicate significant positive gene-receptor correspondence ($p < 0.05$ against a null distribution of surrogate cortical maps with preserved spatial autocorrelation, after applying FDR correction across layers and across all genes matched with the same receptor); grey cells indicate no significance after FDR correction. Columns indicate cortical layers, and rows indicate gene-receptor pairs.(**d**) Summary of the proportion of FDR-corrected significant correlations from a-c, for each layer and each broad receptor type.

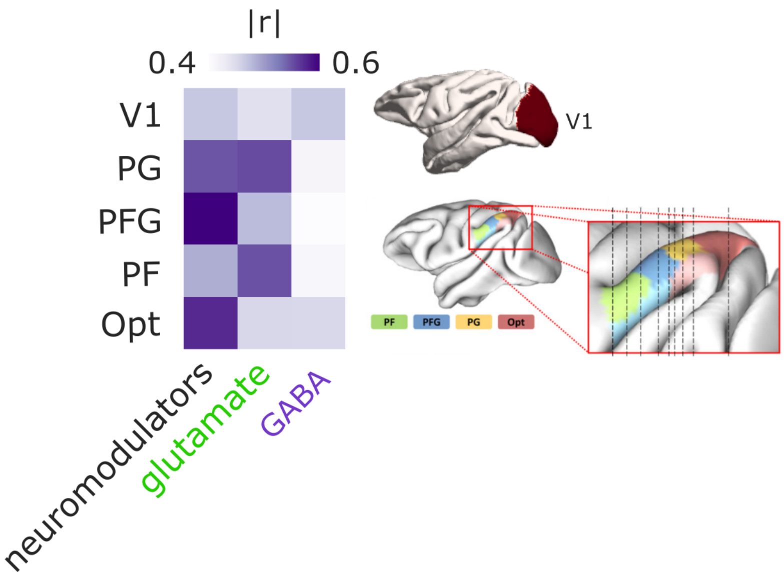

Figure S13. **Gene-receptor correlations across layers |** Average magnitude of correlation between gene expression and receptor density across cortical layers 1 to 6 (each layer is one data-point), for macaque primary visual area V1 and four sub-regions of the macaque inferior parietal lobe (PF, PFG, PG, Opt). Results are shown separately for glutamate receptors, GABA receptors, and neuromodulatory receptors (serotonergic, noradrenergic, cholinergic, and dopaminergic).

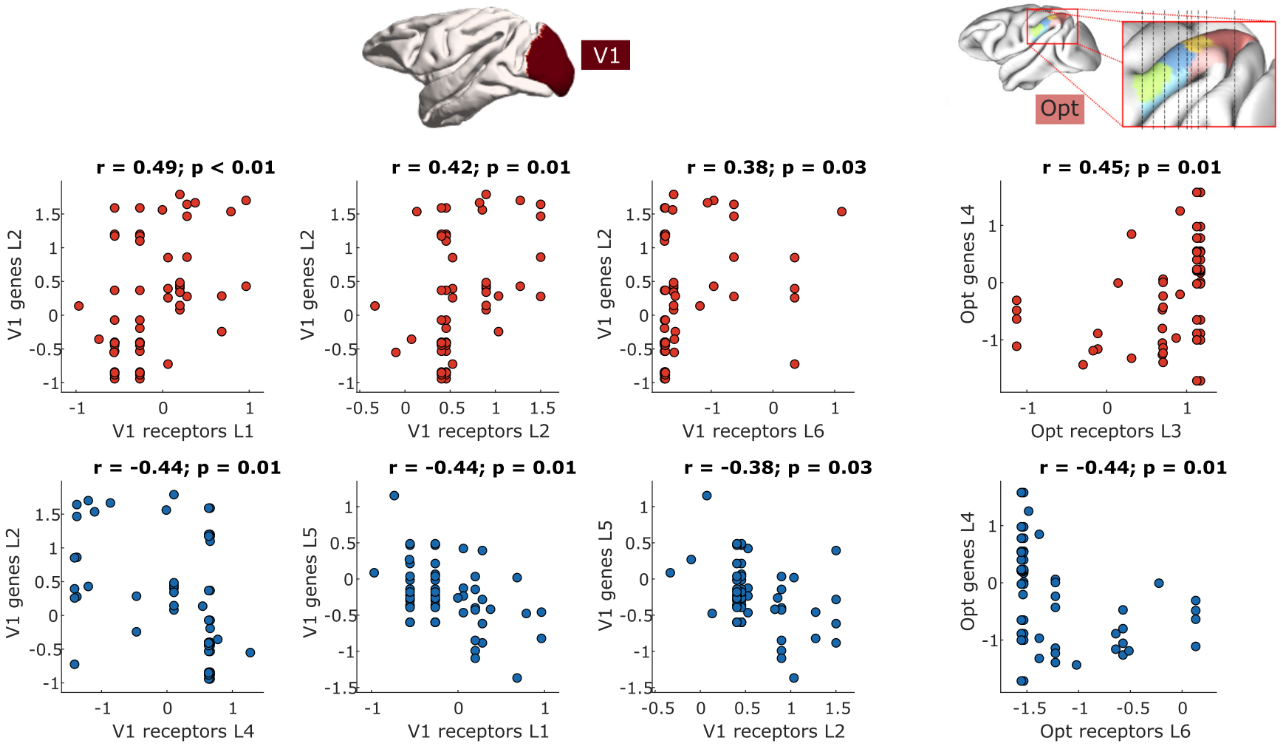

Figure S14. **Correlations of gene and receptor expression in different layers |** Significant (FDR-corrected) correlations between relative gene expression and relative receptor density across layers. No significant correlations were found after FDR correction for PF, PFG and PG sub-regions of IPL, so only V1 and Opt are shown. Each data-point represents one gene-receptor pair. Data are z-scored.

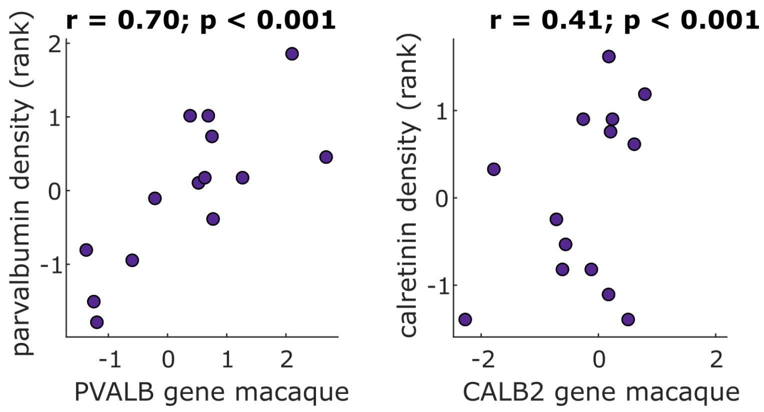

Figure S15. **Region-by-region correspondence of macaque parvalbumin- and calretinin-immunoreactive neurons with gene expression, for a subset of visual, auditory, and somatosensory areas |** Left: regional rank of parvalbumin-immunoreactive neurons versus regional *PVALB* gene expression. Right: regional rank of calretinin-immunoreactive neurons versus regional *CALB2* gene expression. Indigo scatter plots indicate significant region-by-region correspondence (Spearman's r, $p < 0.05$ against a null distribution of surrogate cortical maps with preserved spatial autocorrelation). Values are z-scored.

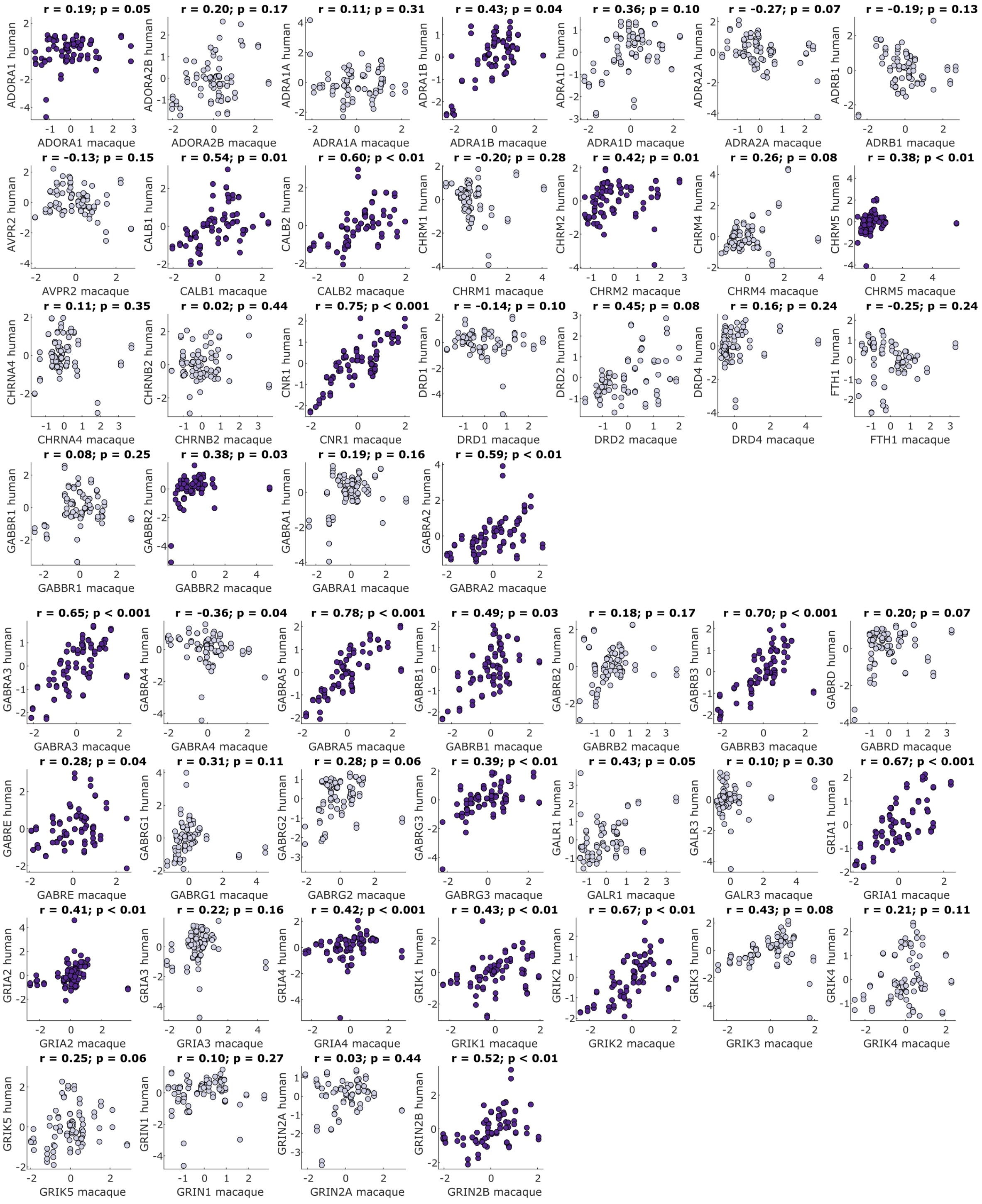

Figure S16. **Region-by-region correspondence between human gene expression from microarray data and macaque gene expression from stereo-seq, for all brain-related genes considered (1/2).** Indigo scatter plots indicate significant ($p < 0.05$) human-macaque correspondence. Gene expression is z-scored.

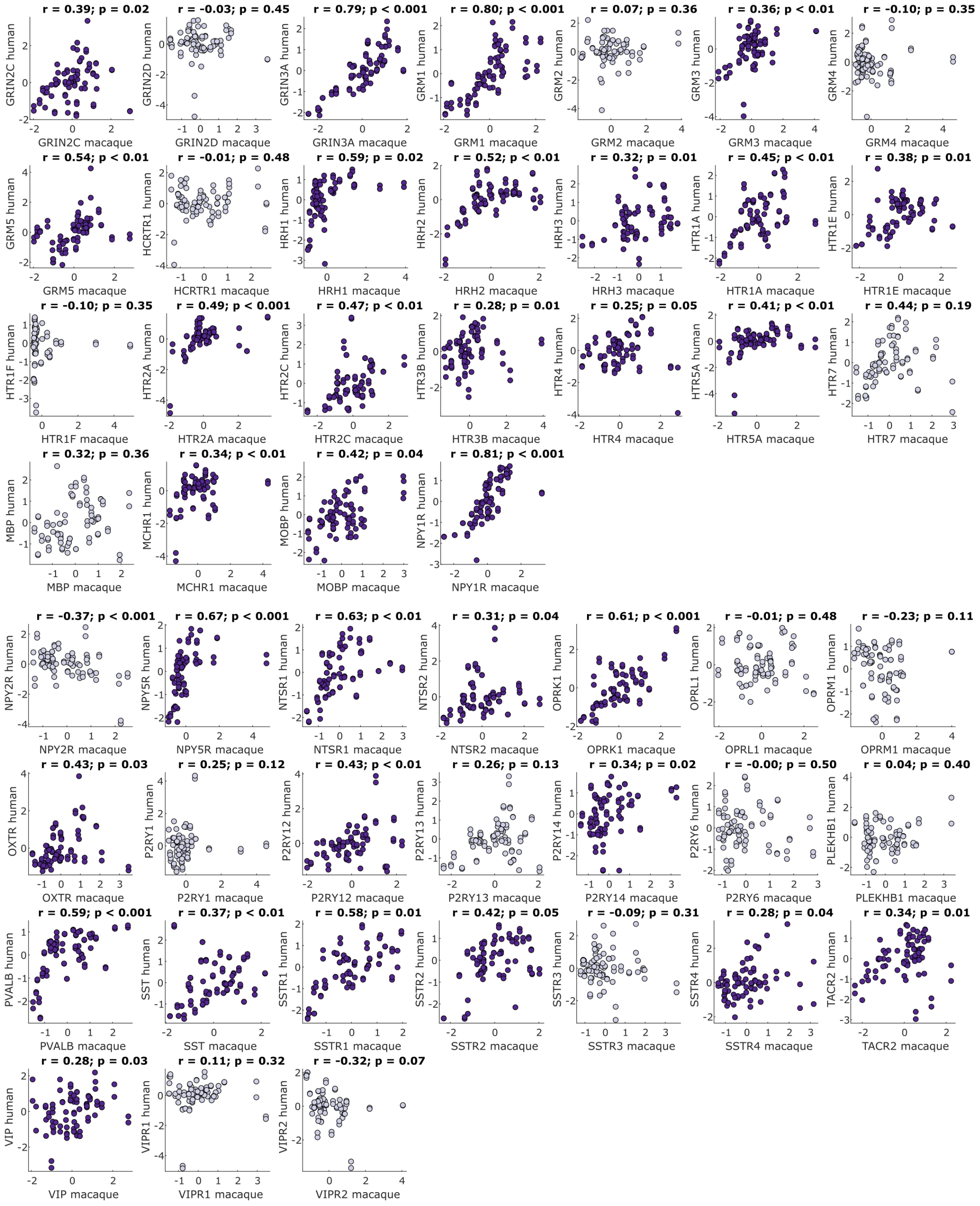

Figure S17. **Region-by-region correspondence between human gene expression from microarray data and macaque gene expression from stereo-seq, for all brain-related genes considered (2/2).** Indigo scatter plots indicate significant ($p < 0.05$) human-macaque correspondence. Gene expression is z-scored.

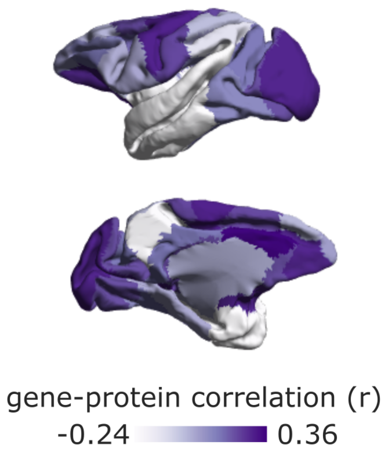

Figure S18. **Regional distribution of the correlation between macaque gene expression and macaque protein density (receptors, T1w:T2w ratio, parvalbumin and calretinin) |** Color of each region indicates the Spearman correlation between macaque gene expression and protein density for that region, across genes-protein pairs. Temporal cortices are not included because receptor density data are not available.

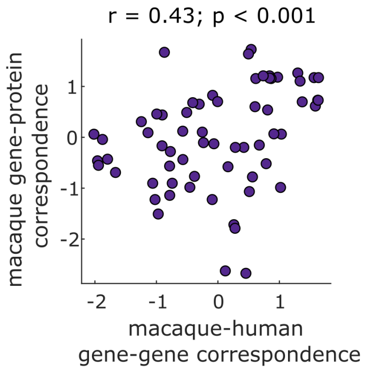

Figure S19. **Regional inter-species gene-gene correspondence recapitulates regional within-species gene-protein correspondence |** Abscissa: regional correlation between macaque gene expression and human gene expression. Ordinate: regional correlation between macaque gene expression and macaque protein density. Spearman's $r = 0.43, p < 0.001$ against a null distribution of surrogate cortical maps with preserved spatial autocorrelation. Each data-point is a region of the Regional Mapping macaque cortical atlas. Values are z-scored.

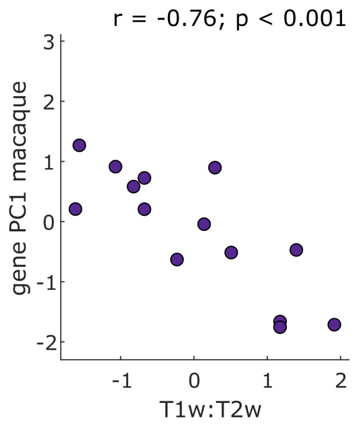

Figure S20. **Replication of region-by-region correspondence between macaque gene PC1 and intracortical myelination |** Abscissa: intracortical myelination (T1w:T2w ratio) from (42). Ordinate: macaque gene PC1. Values are z-scored. Significance of correlation is assessed against a null distribution of surrogate cortical maps with preserved spatial autocorrelation.

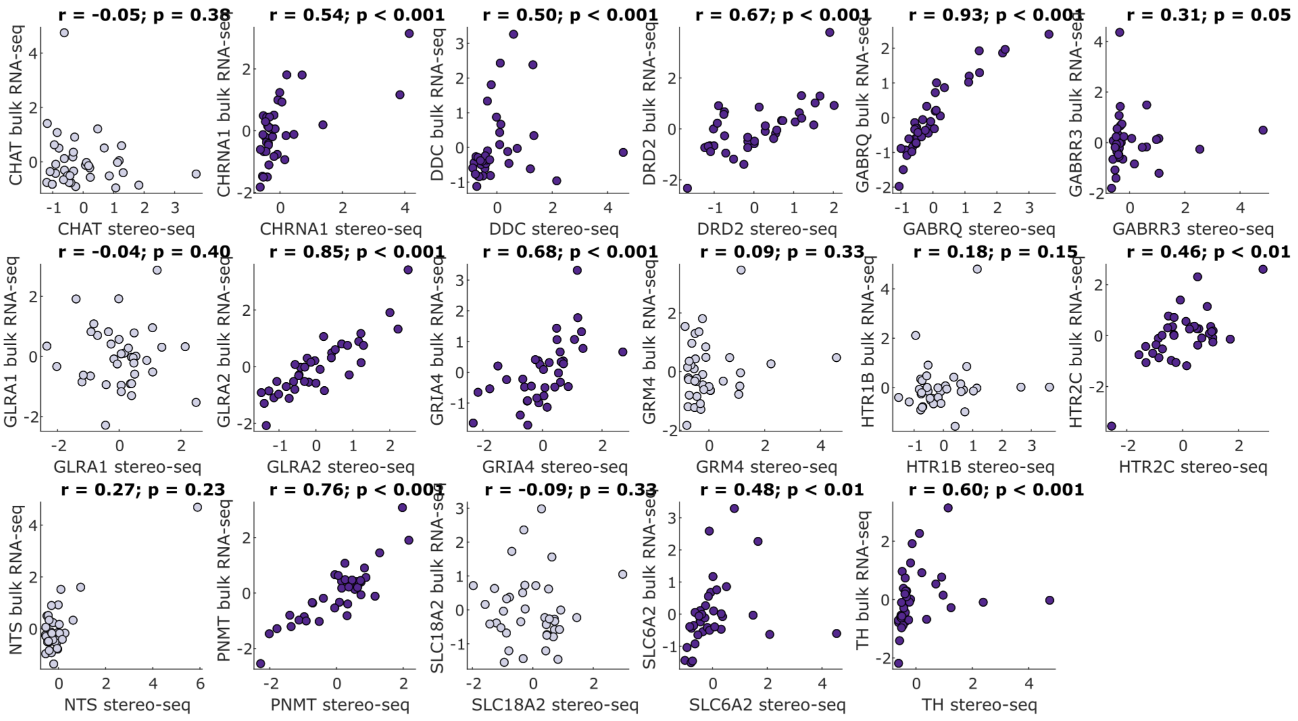

Figure S21. **Region-by-region correspondence between macaque gene expression datasets |** Abscissa: macaque gene expression from stereo-seq (16). Ordinate: macaque gene expression from RNA-seq (17). Indigo scatter plots indicate significant ($p < 0.05$) correspondence. Gene expression is z-scored.

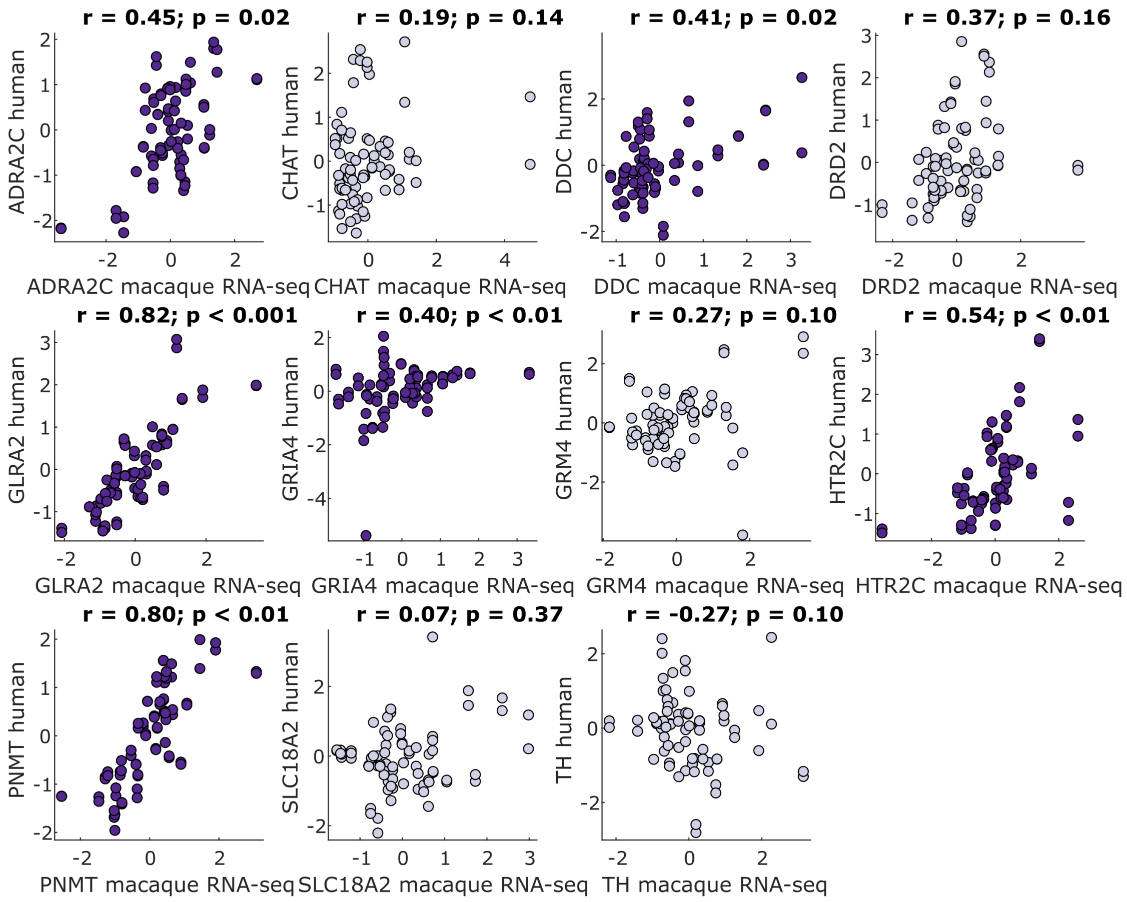

Figure S22. **Region-by-region correspondence between human gene expression from AHBA microarray and macaque gene expression from RNA-seq |** Abscissa: human gene expression from microarray data (15). Ordinate: macaque gene expression from RNA-seq (17). Indigo scatter plots indicate significant ($p < 0.05$) human-macaque correspondence. Gene expression is z-scored. See (fig. S24) for results after FDR adjustment applied across all genes that are being compared against the same receptor.

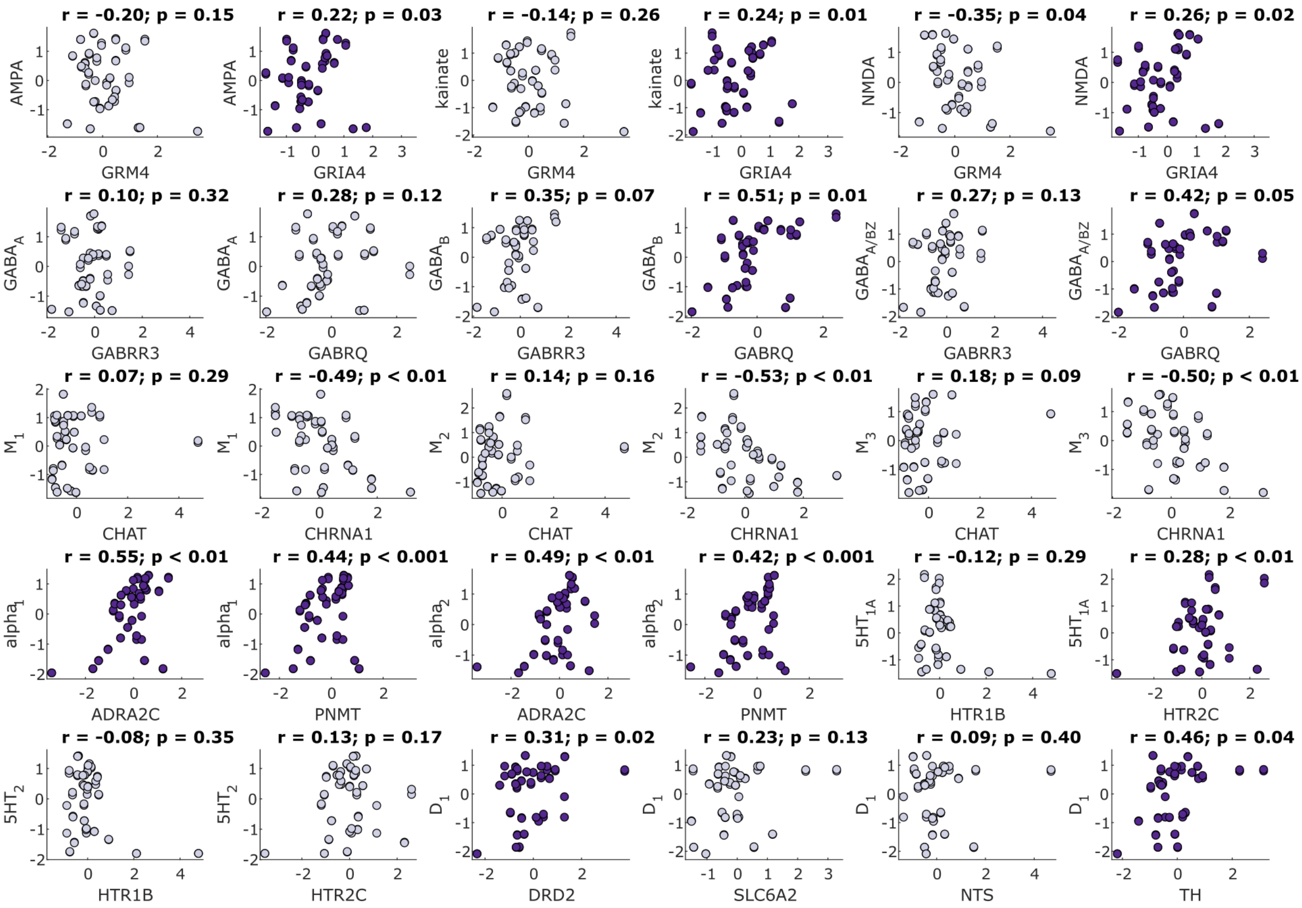

Figure S23. **Region-by-region correspondence between macaque receptor density from autoradiography and macaque gene expression from RNA-seq |** Abscissa: macaque receptor density from autoradiography (39). Ordinate: macaque gene expression from RNA-seq (17). Indigo scatter plots indicate significant ($p < 0.05$) gene-receptor correspondence. Values are z-scored.

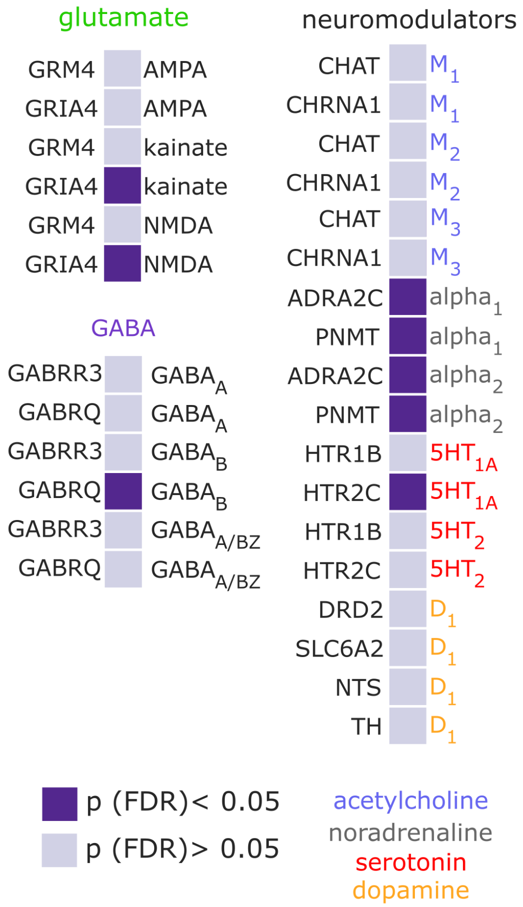

Figure S24. **Significance of correlations between macaque cortical receptor density from autoradiography and gene expression from bulk RNA-seq, after FDR adjustment for multiple comparisons |** Indigo cells indicate significant correlation across regions (Spearman's r, $p < 0.05$ against a null distribution of surrogate cortical maps with preserved spatial autocorrelation), after applying FDR correction for multiple comparisons across all genes matched with the same receptor.

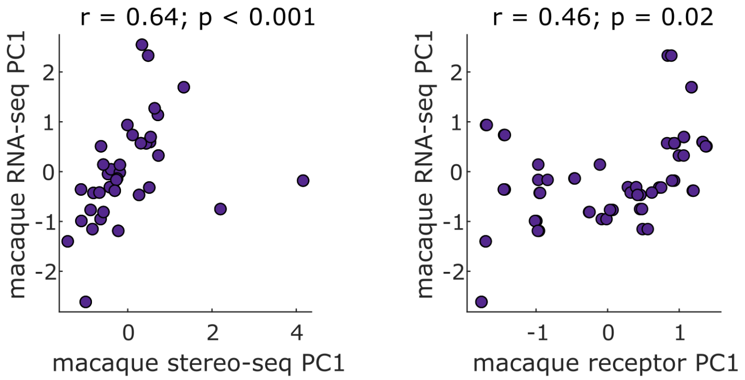

Figure S25. **Region-by-region correspondence of macaque gene PC1 from RNA-seq, with macaque gene PC1 from stereo-seq and macaque receptor PC1 |** (**Left**) Abscissa: macaque gene PC1 from stereo-seq. Ordinate: macaque gene PC1 from RNA-seq. (**Right**) Abscissa: macaque receptor PC1. Ordinate: macaque gene PC1 from RNA-seq. Values are z-scored. Significance of correlation is assessed against a null distribution of surrogate cortical maps with preserved spatial autocorrelation.

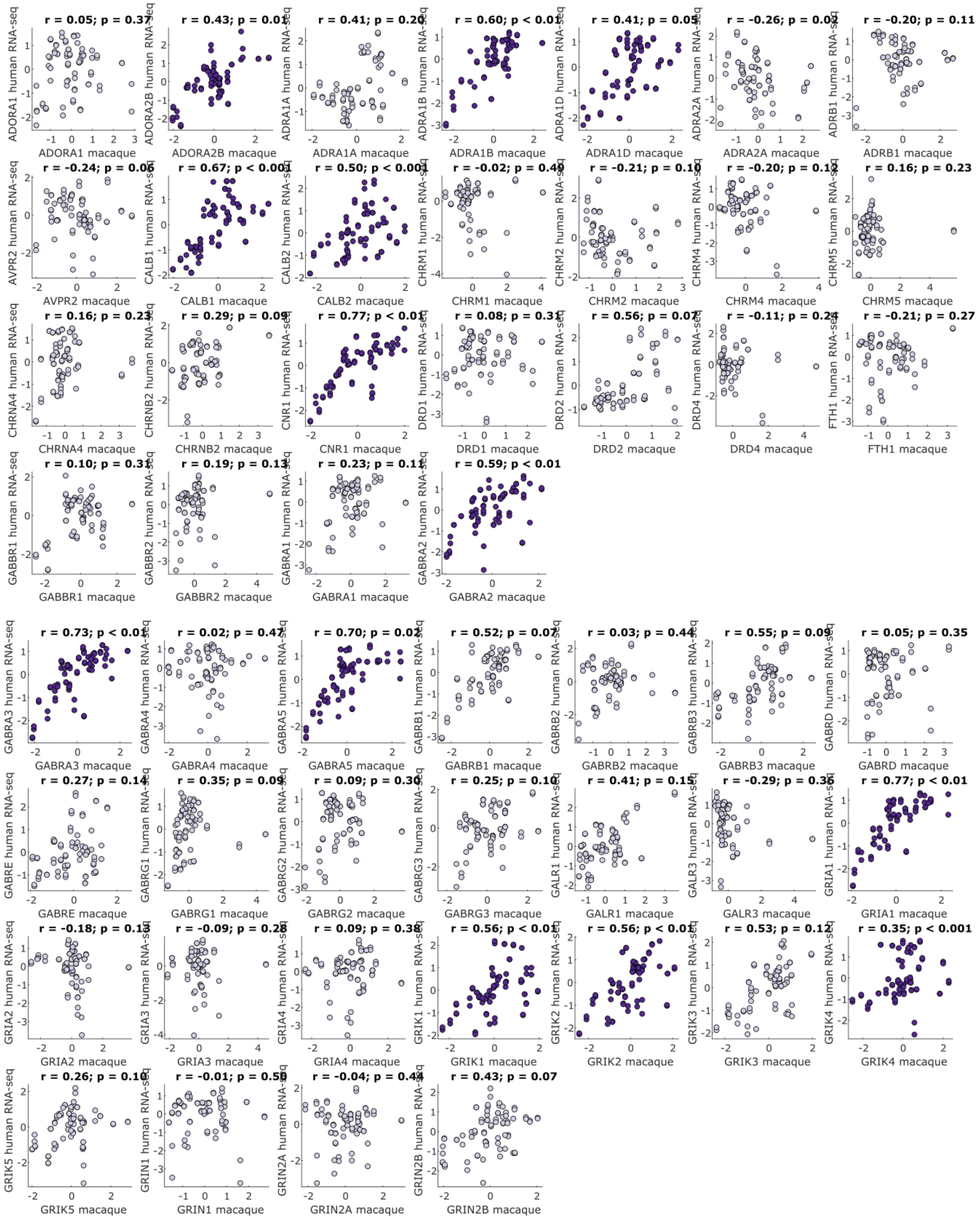

Figure S26. **Region-by-region correspondence between human gene expression from RNA-seq and macaque gene expression from stereo-seq (1/2) |** Indigo scatter plots indicate significant ($p < 0.05$) human-macaque correspondence. Gene expression is z-scored.

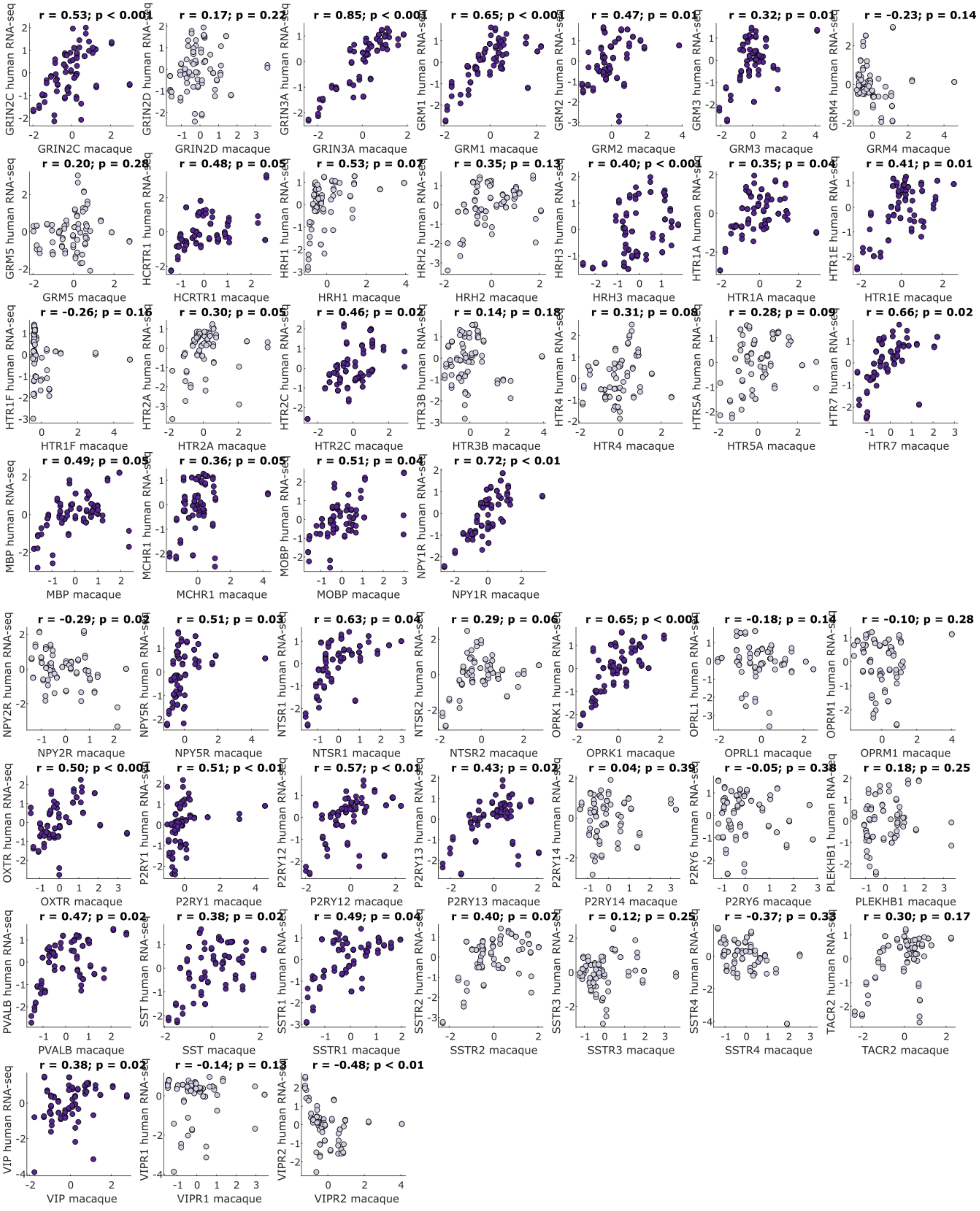

Figure S27. **Region-by-region correspondence between human gene expression from RNA-seq and macaque gene expression from stereo-seq (2/2) |** Indigo scatter plots indicate significant ($p < 0.05$) human-macaque correspondence. Gene expression is z-scored.

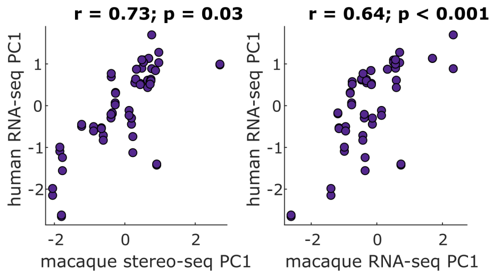

Figure S28. **Region-by-region correspondence of human gene PC1 from RNA-seq, with macaque gene PC1 from stereo-seq and macaque gene PC1 from RNA-seq |** (**Left**) Abscissa: macaque gene PC1 from stereo-seq. Ordinate: human gene PC1 from RNA-seq. (**Right**) Abscissa: macaque gene PC1 from bulk RNA-seq. Ordinate: human gene PC1 from RNA-seq. Values are z-scored. Significance of correlation is assessed against a null distribution of surrogate cortical maps with preserved spatial autocorrelation.

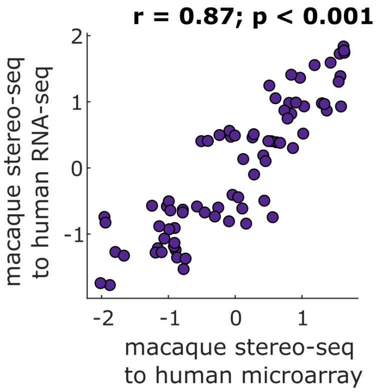

Figure S29. **The pattern of regional inter-species correlation of gene expression is recapitulated when using human RNA-seq data |** Abscissa: regional inter-species correlation of gene expression, using macaque stereo-seq and human microarray data. Ordinate: regional inter-species correlation of gene expression, using macaque stereo-seq and human RNA-seq data. Significance of correlation is assessed against a null distribution of surrogate cortical maps with preserved spatial autocorrelation.

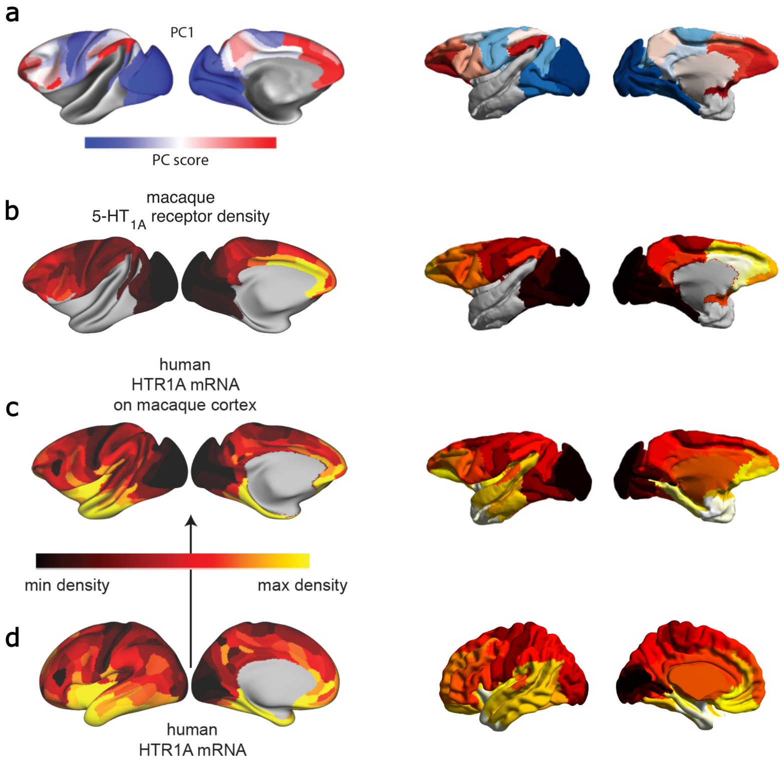

Figure S30. **Mapping macaque receptor autoradiography and human gene expression data to the Regional Mapping parcellation |** (**a**) PC1 of macaque receptor density from autoradiography, in the original parcellation from (39) (left) and mapped onto the macaque Regional Mapping atlas of (55) (right). (**b**) Macaque *5HT$_{1A}$* receptor density, in the original parcellation from (39) (left) and mapped onto the macaque Regional Mapping atlas of (55) (right). (**c**) Human *HTR1A* mRNA mapped onto the macaque parcellation by (39) (left) and mapped onto the macaque Regional Mapping atlas of (55) (right). (**d**) Human *HTR1A* mRNA, mapped on the HCP-MMP human cortical parcellation by (39) (left) and mapped onto the human version of the Regional Mapping atlas made by (57) (right). Panels a-d are modified from Figures 2a, 7c, and 7f of (39), published under CC-BY 4.0 license: http://creativecommons.org/licenses/by/4.0/.
